# “Patient's Family Wants an Update”: A Curriculum for Senior Medical Students to Deliver Telephone Updates for Hospitalized Patients

**DOI:** 10.15766/mep_2374-8265.11256

**Published:** 2022-05-20

**Authors:** Christopher J. Edwards, James T. Fitzgerald, Lauren A. Heidemann

**Affiliations:** 1 Third-Year Resident, Department of Internal Medicine, University of Michigan; 2 Professor Emeritus of Learning Health Sciences, University of Michigan Medical School; 3 Associate Professor, Department of Internal Medicine, University of Michigan Medical School

**Keywords:** Communication Skills, Curriculum Development, Hospital Medicine, Internal Medicine, Role-Play/Dramatization, Self-Assessment

## Abstract

**Introduction:**

Residents have the important task of updating family members of hospitalized patients, often by telephone. There are limited curricula dedicated to preparing medical students for this task, which will become their responsibility as residents.

**Methods:**

We created a virtual workshop, including four patient cases, to facilitate teaching senior medical students enrolled in an internal medicine residency preparation course. Students alternated role-playing either physician or family member. We assessed performance using a self-assessment rubric before (preworkshop) and after (postworkshop) a didactic session. We compared pre- and postworkshop scores using *t* tests. We also used a retrospective pre-post survey with a 5-point Likert scale to assess each participant's comfort level, knowledge, and perceived ability.

**Results:**

Thirty-nine students completed the pre- and postworkshop evaluation (response rate: 70%). The mean score on the preworkshop self-assessment was 83% (*SD* = 9%) and on the postworkshop self-assessment was 94% (*SD* = 8%; *p* < .01), with a large effect size of 1.22. Among the 31 students (62%) who completed the survey, there was improvement in comfort level (2.9 vs. 3.7, *p* < .001), knowledge (2.7 vs. 3.8, *p* < .001), and perceived ability (2.9 vs. 3.7, *p* < .001).

**Discussion:**

Our workshop was effective in teaching medical students a structured format for providing telephone updates and was well received. The workshop was also effective when delivered virtually (with videos off) to mimic the non-face-to-face communication that occurs when delivering family updates by telephone. The curriculum could be expanded to other learner groups.

## Educational Objectives

By the end of this workshop, learners will be able to:

1. Incorporate key components of a telephone update when calling hospitalized patients’ families.
2. Comfortably provide telephone updates and address the common challenges that arise, including the need for empathy, honesty, and setting boundaries.

## Introduction

One of the key responsibilities of a physician is to provide medical updates to the family members of hospitalized patients. This regular communication is important for establishing a therapeutic relationship, gaining deeper understanding of the patient's history and current circumstances, and facilitating shared decision-making between the physician, the patient, and the patient's loved ones. Both patients and their family members perceive the involvement of a family member to improve overall quality of care.^1^ Although medical updates are often completed face to face at bedside, many times the only means of communication is via telephone call due to geographical barriers, time constraints, and, more recently, restricted visitor policies due to the COVID-19 pandemic.^2^ These telephone updates produce additional challenges, such as a lack of nonverbal communication, that physicians must overcome.^3,4^

There is a significant emphasis on development and assessment of effective communication skills throughout medical school training, and multiple curricula address telephone triage and making calls in the outpatient setting, including recent *MedEdPORTAL* publications by Roth, Lane, and Friedman and McDaniel and colleagues.^5–10^ However, there are far fewer structured educational materials devoted to communicating with families and loved ones or providing updates for hospitalized inpatients. Yet, from day one, resident physicians are expected to update family members by telephone, often without attending supervision.^11^ While many of the principles of effective communication are similar between the ambulatory and inpatient setting, there are several unique challenges in the inpatient setting, including that (1) physicians often do not have an established relationship with the patient/family, (2) hospitalized patients often are sicker and may have many more medical issues that require discussion, and (3) many times, these telephone updates must be performed while simultaneously caring for and triaging other acutely ill hospitalized patients. There are many commonly used communication techniques that are helpful when interacting with patients or their families regardless of the setting. These include protocols such as SPIKES (setting, perception, invitation, knowledge, emotion, and strategy and summary) for delivering bad news or NURSE (naming, understanding, respecting, supporting, and exploring) for empathetic communication.^12^ Several similarities exist between our checklists and these communication techniques. For example, preparing in advance, exploring understanding, avoiding jargon, allowing for questions, and summarizing information are key features in both these previously published frameworks as well as in our own curriculum.

Medical training programs have increasingly used simulated learning encounters to allow for safe practice of clinical skills outside the real, clinical environment. Simulated learning allows for immersive practice and has been demonstrated to boost trainee confidence.^13^ Simulation is beneficial not just for technical or procedural skills but for interpersonal communication skills as well.^14^ One such method of simulation is peer role-play, where one student plays the role of the physician and the other plays the role of a patient. A systematic review of peer role-play has shown it to be effective at improving communication skills, well appreciated by participants, and less costly than using paid standardized patient actors.^15^

To help prepare senior medical students to deliver family updates over the telephone for hospitalized patients, we created a virtual curriculum using a peer role-playing exercise. We used principles of adult learning theory to guide the design of our curriculum.^16^ We targeted learners with a high intrinsic level of motivation (medical students preparing to take on new responsibilities) and incorporated experiential learning (learning from mistakes) and formative assessment to guide their learning.

## Methods

### Authors

The authors included an internal medicine resident (Christopher J. Edwards) and an academic hospitalist (Lauren A. Heidemann), who both had experience in providing updates to family members of hospitalized patients and great interest in improving this process. In addition, Lauren A. Heidemann codirected the medical school's residency preparation course curriculum and was also director of a multistation objective structured clinical examination for senior medical students assessing the effectiveness of physician-patient communication skills.

### Development

We constructed a curriculum consisting of a two-page Family Update Guide and a complementary PowerPoint presentation (Appendices A and B). These provided a structured framework for supplying complete yet efficient telephone updates. We created four unique patient cases involving family updates to facilitate interactive practice sessions (Appendix C). All materials were reviewed by multiple internal medicine faculty physicians at the University of Michigan. We then used these materials in a virtual workshop as described below.

### Setting and Participants

The participants were 56 senior medical students at the University of Michigan Medical School enrolled in an internal medicine residency preparation course in the spring of 2021. This 4-week-long course ran twice during the spring and consisted of a variety of didactics, simulations, and role-plays covering a broad range of topics to help ease the transition between medical school and intern year.^17^ In 2021, all didactics and small groups were administered in the virtual setting due to the COVID-19 pandemic.

### Intervention

We facilitated two virtual workshops, each with a different group of fourth-year medical students. The first session lasted 60 minutes, and the second session lasted 75 minutes, lengthened based on feedback from first session to allow for longer debrief/reflection. The workshop used four fictional cases (cases A, B, C, and D; Appendix C), each involving a hospitalized patient and a family member who needed to be updated by telephone. Each interactive case required two participants: One participant played the role of physician providing a telephone update, and the other participant played the role of the family member. We designed all cases based on common internal medicine diagnoses. Each case outlined clear expectations for each participant's task, consistent with published practical guidelines for effective peer role-play.^18^ Each case was accompanied by specific instructions for the two roles. The physician instructions consisted of the patient's name and demographics, history of presenting illness, interval events, physical examination, laboratory data, and imaging data. The family member instructions consisted of a simple understanding of the chief complaint, last update received, some social background, and a short list of questions that were to be asked of the physician at some point during the encounter. The questions asked by the family member were designed to meet one of the following goals: (1) prompt an opportunity for empathetic response from the physician, (2) challenge the physician to decide whether a boundary needed to be set (e.g., asking for an exception to a no-visitor policy), and (3) ask the physician to provide an honest response even when it meant sharing difficult news.

We randomly assigned all workshop participants to a partner and gave them a randomized predetermined order in which to work through the four cases. After starting with a brief introduction, we split participants into private virtual rooms using Zoom videoconferencing software and asked them to practice giving family updates for two cases, with each participant playing the role of physician for one case and the role of family member for the other case. We instructed the participants to treat the situation as a real telephone update and to provide all the information they felt was relevant for a less-than-10-minute telephone update. Video cameras were turned off during this time to better simulate the audio-only aspect of telephone calls. After both cases had been completed, participants scored themselves using the preworkshop self-assessment described below (Appendix D).

Following the preworkshop component, participants rejoined as a group. Next, we conducted a 20-minute didactic session using the previously mentioned PowerPoint presentation and two-page Family Update Guide (Appendices A and B).

After the didactic presentation and a brief open question-and-answer period, participants completed the postworkshop component of the lesson. This was conducted similarly as before: Participants paired with the same partner, and each took a turn as the physician using the remaining two cases. We asked the participants to grade themselves using the postworkshop self-assessment, similar to the previous assessment. The remainder of the session was used to debrief in open dialogue about lessons learned during the workshop. See the Figure for a visual summary of workshop events.

**Figure.**
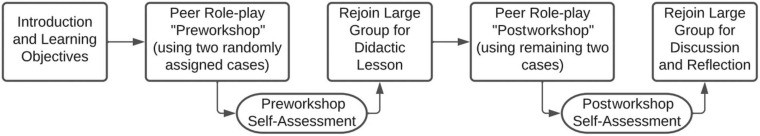
Timeline for workshop on providing telephone updates to a hospitalized patient's family member.

### Family Update Guide

The Family Update Guide we created (Appendix A) was reviewed by three content experts (clinical faculty in hospital medicine). Simultaneous with the development of this guide, a separate guideline, based upon multidisciplinary consensus, was published that highlighted many of the same important steps and techniques for updating family.^19^ This provided further support for the content validity of our guide.

### Assessments

All participants completed a self-assessment in the electronic survey platform Qualtrics, which consisted of a 15-item checklist (Appendix D). Checklist items included confirming the identity of the telephone call recipient, properly introducing oneself and one's role on the medical team, asking for permission to proceed with an update, assessing the family member's current understanding of the situation, providing an overall assessment of the patient, adequately addressing all relevant medical facts (case-specific), outlining the medical plan, excluding unnecessary minor details, confirming that the family member understood the information, allowing questions to be asked, properly concluding the call with clear expectations of time frame for future updates, and refraining from using jargon. Additionally, each case was accompanied by three specific questions to be asked by the family member, as described above. Each checklist item scored 1 point, for a maximum score of 15. In the rare cases where the family member failed to ask one of the three specific questions, the total score denominator was lessened by 1.

Students were included in statistical analysis only if they completed both the preworkshop and postworkshop cases and the self-assessments.

### Curriculum Evaluation

Immediately after the workshop, participants were invited to complete an online evaluation of the curriculum (Appendix E). A retrospective pre-post design was used for this survey.^20^ Participants rated comfort level on ability to provide family updates using a 5-point Likert scale (1 = *very uncomfortable,* 2 = *uncomfortable,* 3 = *neutral,* 4 = *comfortable,* 5 = *very comfortable*). They were also asked to rate their knowledge level and personal ability to provide family updates before and after the workshop (1 = *very poor,* 2 = *poor,* 3 = *fair,* 4 = *good,* 5 = *very good*). Additionally, they were asked two dichotomous (yes/no) questions: (1) Do you feel the session was helpful in preparing you for intern year? (2) Do you plan to use the information presented in the future?

### Statistics

Differences between preworkshop and postworkshop self-assessment scores were determined by *t* test (two-tailed) as scores were anonymous, that is, unpaired. Case difficulty differences were examined for both preworkshop and postworkshop scores using analyses of variance (*p* = .05). Differences in the postsession survey scores (i.e., differences in the retrospective pre-post scores) were examined by paired *t* tests (two-tailed). Magnitude of the intervention was examined by effect size, with a measure of equal to or greater than 0.33 indicating a significant effect in educational research.^21^ All analyses were completed in JMP Pro 15.2.0 (SAS Institute).

This intervention was determined exempt by the University of Michigan Institutional Review Board (HUM00194296).

## Results

### Preworkshop and Postworkshop Self-Assessments

Fifty-six medical students were enrolled in the internal medicine residency preparation course, of whom 39 completed both the pre- and postworkshop self-evaluations (response rate: 70%).

The average total score on the preworkshop self-assessment was 83% (*SD* = 9%) and on the postworkshop self-assessment was 94% (*SD* = 8%; *p* < .01). The effect size was 1.22, which represents a large effect size for an educational intervention. The four self-assessment items that demonstrated most improvement between assessments were asking permission to give an update (64% pre, 92% post), summarizing the overall condition/trajectory (74% pre, 95% post), confirming the family member understood information from the update (51% pre, 90% post), and setting clear expectations for when next update would be (67% pre, 90% post).

When analyzing by case, there was a difference in the level of difficulty between cases C and D at preworkshop self-assessment. However, this difference was not indicated at postworkshop self-assessment (results not shown). By this analysis, the remaining cases did not demonstrate a significant difference in difficulty.

### Survey

Thirty-one students (62%) participated in the postsession survey. Ratings of comfort, knowledge, and ability to update patients’ families increased significantly (Table 1), and 100% of participants stated they would use information from this session in the future (Table 2). In an optional short-answer question, multiple participants indicated that they enjoyed the interactive nature of the lesson and found the before-and-after self-assessment format to be helpful in elucidating areas of weakness.

**Table 1.**
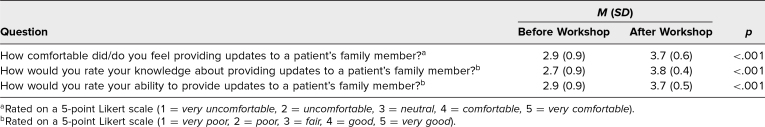
Comfort, Knowledge, and Ability Results From Retrospective Pre-Post Survey (*N* = 31)

**Table 2.**
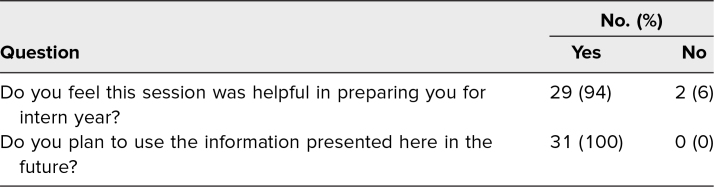
Dichotomous Question Results From Retrospective Pre-Post Survey (*N* = 31)

## Discussion

We designed and implemented a curriculum using a combination of peer role-playing and didactic lecture to prepare fourth-year medical students to give effective telephone updates to family members of hospitalized patients. Learners gradually become more capable of delivering medically accurate updates as their fund of knowledge increases during training. The purpose of this curriculum was to provide an organizational framework for delivering comprehensive family updates upon which learners could build as their medical knowledge expanded. Given significant time constraints during a physician's day on an inpatient team, an emphasis was placed on sharing information concisely and using a structured format to ensure that no key pieces of information were excluded. Learners also practiced navigating real-world challenges such setting boundaries, providing empathetic responses, and sharing difficult facts. Using pre- and postworkshop objective evaluation, we demonstrated that baseline performance deficits were heterogeneous (different students struggled with different aspects of the task) but that the curriculum was effective at increasing competency with telephone updates regardless of baseline performance. Objective scores improved after the didactic session to a statistically significant degree regardless of which randomly assigned order the cases were completed in. Additionally, participants had a positive subjective response to this session as determined by the retrospective pre-post survey.

The virtual setting worked very well for this curriculum as students had cameras turned off and thus could not rely on nonverbal cues, similar to real life. We recommend that if instructors implement this curriculum, they use a similar format, as many students commented it helped to increase the authenticity of the role-play activity.

Several limitations are acknowledged with this project. First, the educational workshop was conducted at a single training program exclusively with fourth-year medical students. Although the intent of the workshop was to help prepare soon-to-be-graduating medical students for their intern year of residency, we cannot say for sure whether the positive results of this education experience are generalizable to trainees at different stages of their education. If resources allow, having a third observer, particularly one who is more experienced and knowledgeable, during the practical portions would offer the added benefits of measuring performance more objectively and providing valuable feedback to augment the learning experience. This could help reduce bias or inaccuracy in self-assessment, which are possible limitations of our evaluation method. Using additional time, perhaps by extending the workshop to 90 minutes, to allow peer-to-peer feedback would also enhance the activity. There are additional challenges common during telephone updates, such as interruptions, encounters with rude or angry relatives, and goals-of-care discussions, that were not explored in this curriculum. In an effort to introduce diversity, names from different ethnic backgrounds were used for the patient cases. We did not assess whether implicit biases may have played a role in the outcomes or scoring of cases. Additionally, it was beyond the scope of this workshop to explore how cultural differences affect sharing of medical information; however, this may be a future area of curricular development.

In conclusion, we have demonstrated that our interactive workshop is an effective tool for teaching medical students a structured approach that covers the key aspects of updating hospitalized patients’ family members. We encourage medical training programs to incorporate this or a similar workshop into their fourth-year medical school curricula. Further work could potentially examine the longer-term impact of the workshop in residency. Additionally, the lesson would benefit from supplementary education about cultural differences in communication practices, as well as tools to practice face-to-face updates.

## Appendices


Family Update Guide.docxFamily Update.pptxPatient Role-Play Cases.docxSelf-Assessment Checklist.docxRetrospective Pre-Post Survey.docx
*All appendices are peer reviewed as integral parts of the Original Publication.*

## References

[1] Di Bernardo V, Grignoli N, Marazia C, Andreotti J, Perren A, Malacrida R. Sharing intimacy in “open” intensive care units. J Crit Care. 2015;30(5):866–870. 10.1016/j.jcrc.2015.05.016 26160723

[2] Boulton AJ, Jordan H, Adams CE, et al. Intensive care unit visiting and family communication during the COVID-19 pandemic: a UK survey. J Intensive Care Soc. Published online April 6, 2021. 10.1177/17511437211007779 PMC9403523 36033248

[3] Marra A, Buonanno P, Vargas M, Iacovazzo C, Ely EW, Servillo G. How COVID-19 pandemic changed our communication with families: losing nonverbal cues. Crit Care. 2020;24:297. 10.1186/s13054-020-03035-w 32503605 PMC7274511

[4] Houchens N, Tipirneni R. Compassionate communication amid the COVID-19 pandemic. J Hosp Med. 2020;15(7):437–439. 10.12788/jhm.3472 32584251

[5] Mack JA, Morgan HK, Fitzgerald JT, Walford EC, Heidemann LA. The development of a video intervention to improve senior medical students’ performance on outpatient telephone encounters: a Delphi analysis and randomized controlled trial. Med Sci Educ. 2021;31(4):1429–1439. 10.1007/s40670-021-01331-w PMC8216674 34178421

[6] Saba GW, Chou CL, Satterfield J, et al. Teaching patient-centered communication skills: a telephone follow-up curriculum for medical students. Med Educ Online. 2014;19(1):22522. 10.3402/meo.v19.22522 24767705 PMC4000921

[7] Roth LT, Lane M, Friedman S. A curriculum to improve pediatric residents’ telephone triage skills. MedEdPORTAL. 2020;16:10993. 10.15766/mep_2374-8265.10993 33117885 PMC7586755

[8] Core competencies for entering medical students. Association of American Medical Colleges. Accessed April 8, 2022. https://www.aamc.org/services/admissions-lifecycle/competencies-entering-medical-students

[9] Edgar L, McLean S, Hogan SO, Hamstra S, Holmboe ES. The Milestones Guidebook: Version 2020. Accreditation Council for Graduate Medical Education; 2020. Accessed April 8, 2022. https://www.acgme.org/portals/0/milestonesguidebook.pdf

[10] McDaniel LM, Molloy M, Hindman DJ, et al. Phone it in: a medical student primer on telemedicine consultation in pediatrics. MedEdPORTAL. 2021;17:11067. 10.15766/mep_2374-8265.11067 33473378 PMC7809927

[11] Raymond MR, Mee J, King A, Haist SA, Winward ML. What new residents do during their initial months of training. Acad Med. 2011;86(10)(suppl):S59–S62. 10.1097/ACM.0b013e31822a70ff 21955771

[12] Berkey FJ, Wiedemer JP, Vithalani ND. Delivering bad or life-altering news. Am Fam Physician. 2018;98(2):99–104. 30215989

[13] Krishnan DG, Keloth AV, Ubedulla S. Pros and cons of simulation in medical education: a review. Int J Med Health Res. 2017;3(6):84–87.

[14] Okuda Y, Bryson EO, DeMaria S Jr, et al. The utility of simulation in medical education: what is the evidence? Mt Sinai J Med. 2009;76(4):330–343. 10.1002/msj.20127 19642147

[15] Gelis A, Cervello S, Rey R, et al. Peer role-play for training communication skills in medical students: a systematic review. Simul Healthc. 2020;15(2):106–111. 10.1097/SIH.0000000000000412 32168292

[16] Taylor DCM, Hamdy H. Adult learning theories: implications for learning and teaching in medical education: AMEE Guide no. 83. Med Teach. 2013;35(11):e1561–e1572. 10.3109/0142159X.2013.828153 24004029

[17] Heidemann LA, Walford E, Mack J, Kolbe M, Morgan HK. Is there a role for internal medicine residency preparation courses in the fourth year curriculum? A single-center experience. J Gen Intern Med. 2018;33(12):2048–2050. 10.1007/s11606-018-4620-6 30094763 PMC6258616

[18] Nestel D, Tierney T. Role-play for medical students learning about communication: guidelines for maximising benefits. BMC Med Educ. 2007;7:3. 10.1186/1472-6920-7-3 17335561 PMC1828731

[19] Mistraletti G, Gristina G, Mascarin S, et al. How to communicate with families living in complete isolation. BMJ Support Palliat Care. Published online October 15, 2020. 10.1136/bmjspcare-2020-002633 33060189

[20] Bhanji F, Gottesman R, de Grave W, Steinert Y, Winer LR. The retrospective pre–post: a practical method to evaluate learning from an educational program. Acad Emerg Med. 2012;19(2):189–194. 10.1111/j.1553-2712.2011.01270.x 22320369

[21] Isaac S, Michael WB. Handbook in Research and Evaluation: A Collection of Principles, Methods, and Strategies Useful in the Planning, Design, and Evaluation of Studies in Education and the Behavioral Sciences. 3rd ed. EdITS Publishers; 1995:95.

