# Supplementary material for: “Patient's Family Wants an Update”: A Curriculum for Senior Medical Students to Deliver Telephone Updates for Hospitalized Patients
Source: MedEdPORTAL. 2022 May 20;18:11256. doi: 10.15766/mep_2374-8265.11256 (PMC9120304; doi:10.15766/mep_2374-8265.11256)
Supplement: Supplementary file 1 — Family Update Guide.docxFamily Update.pptxPatient Role-Play Cases.docxSelf-Assessment Checklist.docxRetrospective Pre-Post Survey.docx [file mep_2374-8265.11256-s001.zip › C. Patient Role-play Cases.docx]

**CASE “A” – PHYSICIAN VERSION**

You are the intern taking care of Bernard Miller, a 68M with obesity, type II diabetes, and hypertension. He lives with his wife Martha. He has had weeks of progressive dyspnea on exertion, orthopnea, swelling in legs, and weight gain. You admitted him from the ED yesterday, and met his wife Martha there. She comments that he avoids going to doctors and occasionally has chest pain that he has not sought treatment for. Since admission, a transthoracic echocardiogram has revealed a newly reduced ejection fraction of 30-35%. He has been started on IV diuretics. Prior to discharge, your team plans to do an ischemic evaluation and start him on appropriate medications for heart failure.

On rounds, while speaking to Bernard, you briefly explained the diagnosis of congestive heart failure and the treatment plan. You are about to call his wife Martha to give her updates.

**Additional Information:**

Vitals: Temp 98.5^o^F, HR 90 (normal sinus), RR 20, BP 134/64, spO2 92% on 2L (stable requirement since ED). He is net negative 1.5L volume in response to two doses of IV Lasix

Physical exam: no acute distress, no murmurs, positive JVD, bibasilar crackles, grossly volume overloaded, warm extremities

Labs: WBC 7, HGB 13, PLT 200

NA 133, K 4.2, BUN 24, CR 0.9 (baseline 0.9)

VBG pH 7.36, Lactate 0.7

BNP 755

hsTrop 15 -> 14

D-dimer within normal limits

Imaging:

EKG with no acute ischemic findings but suggestive of prior anterior infarct

Chest X-ray suggestive of cardiomegaly and pulmonary edema with no consolidation

Transthoracic echo with LV EF 30-35% and anterior wall motion abnormalities, no significant valve dysfunction

**CASE “A” – PATIENT’S WIFE (“MARTHA MILLER”)**

You are the wife of Bernard Miller, a 68M with obesity, type II diabetes, and hypertension. He rarely goes to the doctor. He occasionally has chest pain but refuses to be evaluated. For the past few weeks, he has been short of breath with exertion, while laying down, and has had a lot of swelling in his legs with weight gain. You finally convinced him to go to the ED yesterday where you met the admitting intern.

Earlier this morning, Bernard called you and said “they told me my heart is failing.” You are very anxious and want to speak with the doctor, who is just about to give you a call.

Use the following as a reference as you roleplay as Bernard’s wife Martha. Try to avoid interruptions. Answer with just “hello.”

**CURRENT UNDERSTANDING**: You have not heard anything from doctors since he was in the ED. You spoke with Bernard two hours ago who said “my heart is failing.” This made you extremely anxious and are afraid that he is about to die.

**QUESTIONS YOU HAVE:**

Please ask/say each of the following at some point during the encounter.

1. “Is he going to have this problem forever?”
2. “I feel so bad, he has been feeling unwell for weeks. I should have tried harder to convince him to come sooner.” – Pause briefly after to allow for an empathetic reply
3. “Can you call me again in a few hours for more updates?”

*You can accept the doctor’s answer.*

**CASE “B” – PHYSICIAN VERSION**

You are the intern taking care of Neeraj Kumar, an 82M with obesity, type II diabetes, CKD, mild cognitive impairment, and multiple brief admissions over the past few months for recurrent cellulitis of left lower extremity. He recently moved in with his son Nitin. He was admitted overnight to general medicine after days of profuse watery diarrhea, abdominal discomfort, and lack of appetite at home. Since admission, his stool tested positive for C. Difficile and he was started on oral vancomycin one hour ago. He had 6 watery bowel movements overnight. He was noted to be dehydrated on intake and received one liter of IV fluids for resuscitation but the data below suggests he could use more.

You met the patient for the first time on rounds this morning and he was notably confused and disoriented to place and time. He was AOx3 when he was admitted. You have not spoken with his son Nitin before. You are about to call his son Nitin to give him updates.

Of note, the hospital’s current visitor policy is NO visitors except in extreme circumstances (impending death).

**Additional Information:**

Vitals: Temp 100.5^o^F, HR 98 (normal sinus), RR 16, BP 110/62, spO2 96% on room air

Physical exam: resting comfortably in bed, AOx1 (knows name, does not know where he is, the year, or why he is here) with otherwise normal neurologic examination, cardiopulmonary examination is normal, abdomen is soft and non-distended but mildly tender, mucous membranes appear dry, skin discoloration of left lower leg but no erythema or weeping

Labs: WBC 19, HGB 13.5, PLT 270

NA 137, K 3.1, Bicarb 18, BUN 24, CR 1.7 (baseline 1.2), glucose 88

Lactate 1.1

Stool C. Diff toxin enzyme immunoassay positive

Urinalysis – 5 ketones but otherwise benign

Imaging:

EKG normal sinus rhythm

Abdominal X-ray with no acute findings and no bowel dilation

**CASE “B” – PATIENT’S SON (“NITIN KUMAR”)**

You are the son of Neeraj Kumar, an 82M with obesity, type II diabetes, CKD, mild cognitive impairment, and multiple brief admissions over the past few months for recurrent cellulitis of lower extremity. You moved in with your father two months ago shortly after your mother died following complications from a stroke. He just finished a course of antibiotics for cellulitis and his leg is looking improved. For the past three days, he has had worsening diarrhea with some abdominal discomfort and poor appetite.

You are about to receive a call from the doctor taking care of your father.

Use the following as a reference as you roleplay as Neeraj’s son Nitin. Try to avoid interruptions. Answer with just “hello.”

**CURRENT UNDERSTANDING**: You have not heard any updates since dropping your father off in the emergency department. You have not heard from the doctor currently taking care of him.

**QUESTIONS YOU HAVE:**

Please ask/say each of the following at some point during the encounter.

1. If the doctor brings up altered mental status / delirium:

“That sounds very unlike him. What do you think is causing this?”

1. “Is this condition ever life-threatening?”
2. “My mother just passed away a few months ago at this hospital. He is scared every time he has to be admitted that the same will happen to him.” – Pause briefly after to allow for an empathetic reply
3. At the end: “I am worried about him. I know the policy limits visitation, but could you try to make an exception for me? I think he would do a lot better if I was there.”

*You can accept the doctor’s answer.*

**CASE “C” – PHYSICIAN VERSION**

You are the intern taking care of Cynthia Lin, a 55F with obesity, systemic lupus erythematosus, and hypothyroidism who was admitted for pneumonia two days ago. Her husband Peter has been receiving daily updates by phone (last update yesterday afternoon). The hospital’s current visitor policy restricts his ability to visit. Cynthia has had a productive cough, malaise, headache, and a new 2 to 3L nasal cannula oxygen requirement which had been stable since arrival to the ED. She has been receiving IV antibiotics.

During the afternoon today, Cynthia’s oxygen requirement increased to 4L, and then from 4L to 6L. You have discussed with her that she may soon require escalation of care to the ICU and she is agreeable to this, and she reaffirms that she would want everything done to prolong her life. She asks that you let her husband know what is happening.

You are about to call her husband Peter to give him updates.

**Additional Information:**

Vitals: Temp 99.5^o^F, HR 110 (sinus tachycardia), RR 28, BP 120/64, spO2 91% on 6L

Physical exam: uncomfortable, tachypneic with increased work of breathing, bilateral rhonchi, tachycardic with no murmurs, soft abdomen, warm, diaphoretic, AOx3

Labs: WBC 15, HGB 14.5, PLT 415

NA 141, K 3.7, Bicarb 19, BUN 24, CR 0.6 (baseline 0.6)

ABG on 6L – pH 7.48, CO2 30, O2 62, lactate 1.5

Procalcitonin 2.02 (high)

Respiratory virus panel is negative

Sputum culture is in in the lab, no results yet

Blood cultures no growth to date

Imaging:

EKG – sinus tachycardia

CXR – progressive, diffuse bilateral lung infiltrates, no pneumothorax

**CASE “C” – PATIENT’S HUSBAND (“PETER LIN”)**

You are the husband of Cynthia Lin, a 55F with obesity, systemic lupus erythematosus, and hypothyroidism who was admitted for pneumonia two days ago. She has had a cough, malaise, headache, fever, and has been requiring some supplemental oxygen by nasal cannula since arrival to the emergency department.

You have been receiving daily updates from the doctor but have not yet had an update today. The hospital’s current visitor policy restricts your ability to visit. You are about to receive a call from the doctor taking care of your wife.

Use the following as a reference as you roleplay as Cynthia’s husband Peter. Try to avoid interruptions. Answer with just “hello.”

**CURRENT UNDERSTANDING**: The doctors have told you that her course has been stable and uncomplicated, but she is still requiring oxygen. She texted you earlier this morning and said she is feeling more tired than before, but you have not heard anything from her in over six hours.

**QUESTIONS YOU HAVE:**

Please ask/say each of the following at some point during the encounter.

1. “Do you think she is going to need a ventilator?”
2. “This is so hard being separated while she is suffering. I wish I could be there.” – Pause briefly after to allow for an empathetic reply
3. Towards the end: “If I don’t hear from her in a few hours, is it okay if I call back for a quick update?”

*You can accept the doctor’s answer.*

**CASE “D” – PHYSICIAN VERSION**

You are the intern taking care of Michelle Smith, a 72F lifelong smoker who has rarely sought medical attention. Over the past 6 months she has had an unintentional weight loss of almost 30 lbs. Over the past couple weeks, she has had yellowing of the skin, pale stool, and severe early satiety. For a few days prior to admission, she had been experiencing severe bloating, nausea, and vomiting after all attempts to eat. She presented to the ED yesterday afternoon where a CT scan of the abdomen demonstrated a 3 cm mass in the head of the pancreas as well as additional findings listed below. She was given fluid and electrolyte repletion and her nausea initially improved with IV ondansetron. She was admitted to your service overnight. Her spouse, Kristin Scott, was updated last night and both she and the patient are aware that this likely represents pancreatic cancer.

This morning, Michelle has had worsening nausea and several episodes of emesis despite two anti-emetic medications. You have discussed that placing a nasogastric tube for decompression would likely improve her symptoms. She is hesitant but agrees and requests you update Kristin by phone. Consults to gastroenterology for biopsy/stent(s) as well as to medical oncology and surgery have already been placed and you expect to hear back from these teams soon. There are still many questions to be answered regarding precise diagnosis, staging, prognosis, and available treatment options.

You are about to call her spouse Kristin to give her updates.

**Additional Information:**

Vitals: Temp 98.1^o^F, HR 90, RR 18, BP 110/64, spO2 95% on room air

Physical exam: uncomfortable appearing with muscle wasting, jaundiced, moist mucous membranes, regular rate and rhythm with no murmurs, lungs clear to auscultation bilaterally, abdomen is soft but distended and uncomfortable to palpation

Labs: WBC 11, HGB 12.7, PLT 380

NA 140, K 3.3, Bicarb 30, BUN 11, CR 0.7 (1.1 on admission, baseline 0.6)

AST 42, ALT, 39, ALKP 380, TBIL 8.4

CA19-9 14,530

Imaging:

EKG – NSR, QTc 420

CT Abdomen & Pelvis – 3 cm mass in the head of the pancreas with upstream dilation of common bile duct to 0.9cm and distension of the proximal duodenum and stomach

**CASE “D” – PATIENT’S SPOUSE (“KRISTIN SCOTT”)**

You are the spouse of Michelle Smith, a 72F who is a lifelong smoker and has rarely seen a doctor. For six months she has had poor appetite and unintentional weight loss of 30 lbs. For the past couple weeks, her skin has become yellowed, her stools have become pale, and she has been less and less tolerant of eating or drinking, now with vomiting every time she tries. This prompted her to seek medical attention in the ED yesterday afternoon.

Ten hours ago you spoke with the night doctor who helped admit Michelle to the hospital. You are about to receive a call from the day team doctor taking care of Michelle.

Use the following as a reference as you roleplay as Michelle Smith’s spouse Kristin Scott. Try to avoid interruptions. Answer with just “hello.”

**CURRENT UNDERSTANDING**: The night doctor explained that Kristin was rehydrated with intravenous fluids and a CT scan showed a mass in the pancreas and that this mass is likely cancer. Kristin knows this as well. Her nausea has improved with medications. You were told specialists will be seeing her during the day but you do not know the specifics.

**QUESTIONS YOU HAVE:**

Please ask/say each of the following at some point during the encounter.

1. “What is her prognosis?”
2. Towards the end: “This is all so shocking and so much information to handle. I am trying to take notes, but I just worry I won’t keep up” – Pause briefly after to allow for an empathetic reply
3. Towards the end: “May I have your cell phone number in case I have more questions later?”

*You can accept the doctor’s answer.*
