# Supplementary material for: “Patient's Family Wants an Update”: A Curriculum for Senior Medical Students to Deliver Telephone Updates for Hospitalized Patients
Source: MedEdPORTAL. 2022 May 20;18:11256. doi: 10.15766/mep_2374-8265.11256 (PMC9120304; doi:10.15766/mep_2374-8265.11256)
Supplement: Supplementary file 1 — Family Update Guide.docxFamily Update.pptxPatient Role-Play Cases.docxSelf-Assessment Checklist.docxRetrospective Pre-Post Survey.docx [file mep_2374-8265.11256-s001.zip › D. Self-Assessment Checklist.docx]

**SELF-ASSESSMENT CHECKLIST**

During the workshop, this survey was available online utilizing Qualtrics surveys. It was completed both pre-workshop and post-workshop. Depending on which case was selected, the survey logic would redirect towards a slightly different set of questions that outlined specific examples from each case, when applicable (denoted by * below).

**Q.** Which case did you just complete in the role of the physician? (Case A, B, C, or D)

**Q.** Was this before or after didactic session? Before / After

Please fill out the scoring rubric self-assessment below honestly. The scores here are not attached to your name and will in no way affect your medical student grades. This is purely for the purpose of assessing the effectiveness of this course.

Please indicate which of the following you performed:

**Q1**. Did you confirm that you were speaking with __*__ before you shared any medical information?

Yes/No

(*case A: patient’s wife Martha) (*B: patient's son Nitin) (*C: patient's husband Peter) (*D: patient's spouse Kristin)

**Q2.** Did you introduce yourself AND state your role (physician, intern) on the medical team?

Yes/No

**Q3**. Did you ask for permission to give an update (ex: "is now an okay time for an update?")

Yes/No

**Q4.** Did you assess family member’s current understanding (ex: “what is last update you’ve received?”)

Yes/No

**Q5.** Did you provide an overall assessment of patient which includes condition and trajectory? (improving, stable, critical)

__*__

Yes/No

*For case A, the example provided in question is "Bernard is in stable condition"

*For case B, the example provided in question is "Your father is currently sick with multiple medical problems which can sometimes be serious" or "he has an infection and is dehydrated but vital signs are currently reassuring"

*For case C, the example provided in question is "Cynthia is unfortunately requiring more and more additional oxygen by nose today. Her breathing is worsening and she may soon require an escalation of care to the intensive care unit"

*For case D, the example provided in question is "Michelle is having high symptom burden which is not yet under control and likely has cancer, but long-term prognosis difficult to determine until we do additional work-up"

**Q6.** Did you explain the big picture including all of the following? __*__

Yes/No

*For case A, you explained briefly what heart failure means, why you suspect that as the diagnosis, and that you think this is the cause of his shortness of breath

*For case B, you explained the diagnosis of C Diff infection, discussed dehydration, and discussed delirium

*For case C, you explained that her respiratory status as a result of pneumonia has acutely worsened, you explained that she has been requiring escalating amounts of supplemental oxygen

*For case D, you explained that her nausea/vomiting has been poorly controlled despite medications, she likely has pancreatic cancer causing these symptoms but additional studies (imaging, biopsy) will be needed to understand prognosis and treatment options, Gastroenterology, Surgery, and Oncology have all been consulted and will see her later today

Yes/No

**Q7.** Did you describe the plan moving forward including all of the following? __*__

*For case A, your plan for diuresis to remove extra fluid / improve shortness of breath, plan to introduce heart failure medications, plan to do ischemic evaluation before discharge

*For case B, your plan for oral vancomycin to treat C Diff, plan for continued fluid resuscitation (dehydrated, do not need to specifically discuss AKI, plan for delirium (reorientation, address discomforts)

*For case C, your plan including high likelihood of needing transfer to the intensive care unit for higher levels of oxygen support, okay but not mandatory to discuss unchanged current treatments (IV antibiotics)

*For case D, your plan for nasogastric tube decompression, anticipate more diagnostics over next couple days (such as imaging, endoscopy +/- stent(s), biopsy), follow-up on Gastroenterology, Surgery, and Oncology consults

**Q8.** Did you appropriately exclude unnecessary details? (ex: you did NOT read all of the vital signs and lab results)

Yes/No

**Q9.** Did you confirm that the family member understood the information presented? (ex: repeat-back method, or "does all of that make sense? Can I clarify anything I just said?")

Yes/No

**Q10.** Did you grant permission for the family member to ask additional questions before concluding the phone call?

Yes/No

**Q11. Case A:** When Martha asked "is he going to have this problem forever?" did you honestly explain that it is a chronic condition but can be managed with medicines?

**Q11. Case B:** When Nitin asked "is this condition ever life-threatening?" did you honestly explain that C Diff can have some life-threatening complications but you do not currently see signs of those?

**Q11. Case C:** When Peter asked "do you think she is going to need a ventilator?" did you honestly explain that it remains a possibility if her conditions continues to deteriorate, but she is not currently at that point?

**Q11. Case D:** When Kristin asked "what is her prognosis?" did you honestly explain that it more information is needed (biopsy, staging, consult team input) before a definitive statement can be made on prognosis. Okay to say prognosis is likely poor overall.

Yes/No

**Q12. Case A:** When Martha expressed "I should have tried harder to convince him to come sooner," did you provide an empathetic response?

**Q12. Case B:** When Nitin expressed that his mother just passed away and his father is scared of the same, did you provide an empathetic response?

**Q12. Case C:** When Peter expressed "this is so hard being separated while she is suffering, I wish I could be there," did you provide an empathetic response?

**Q12. Case D:** When Kristin expressed "this is all so shocking and so much information to handle. I am trying to take notes, but I just worry I won't keep up," did you provide an empathetic response?

Yes/No

**Q13. Case A:** When Martha asked "can you call me again in a few hours for more updates?" did you set a boundary that it is NOT necessary to provide another update so soon?

**Q13. Case B:** When Nitin asked for an exception to the visitor policy, did you set a boundary that it is NOT appropriate for an exception to be made in this case?

**Q13. Case C:** When Peter asked "if I don't hear from her in a few hours, is it okay if I call back for a quick update?" did you indicate that this is appropriate in these circumstances?

**Q13. Case D:** When Kristin asked for your cell phone number, did you appropriately set a boundary that this is not appropriate? (ideally would alternatively recommend she call the hospital line)

Yes/No

**Q14.** Did you say goodbye AND set a clear expectation for when next update will be?

Yes/No

**Q15.** Did you refrain from using medical jargon, or, if used, did you immediately explain in simplified terms?

Yes/No

Each “Yes” answer scores 1 point. Maximum 15 points. If one of the questions was unintentionally not asked by the family member and thus is not applicable, subtract this point from the total maximum.
