# Supplementary material for: “Patient's Family Wants an Update”: A Curriculum for Senior Medical Students to Deliver Telephone Updates for Hospitalized Patients
Source: MedEdPORTAL. 2022 May 20;18:11256. doi: 10.15766/mep_2374-8265.11256 (PMC9120304; doi:10.15766/mep_2374-8265.11256)
Supplement: Supplementary file 1 — Family Update Guide.docxFamily Update.pptxPatient Role-Play Cases.docxSelf-Assessment Checklist.docxRetrospective Pre-Post Survey.docx [file mep_2374-8265.11256-s001.zip › E. Retrospective Pre-Post Survey.docx]

**Retrospective Pre- Post Survey for Curriculum Evaluation**

During the workshop, this survey was available online utilizing Qualtrics surveys.

Please fill out the evaluation below honestly. The replies here are not attached to your name and will in no way affect your grades. This is only for the purpose of assessing the need for and effectiveness of this course.

1. Prior to today’s session, how comfortable did you feel providing updates to a patient’s family member?

Very uncomfortable (1), Uncomfortable (2), Neutral (3), Comfortable (4), Very Comfortable (5)

2. After today’s session, how comfortable do you feel providing updates to a patient’s family member?

Very uncomfortable (1), Uncomfortable (2), Neutral (3), Comfortable (4), Very Comfortable (5)

3. Prior to today’s session, how would you rate your knowledge about providing updates to a patient’s family member?

Very poor (1), Poor (2), Fair (3), Good (4), Very Good (5)

**4. After** today’s session, how would you rate your knowledge about providing updates to a patient’s family member?

Very poor (1), Poor (2), Fair (3), Good (4), Very Good (5)

5. Prior to today’s session, how would you rate your ability to provide updates to a patient’s family member?

Very poor (1), Poor (2), Fair (3), Good (4), Very Good (5)

6. After today’s session, how would you rate your ability to provide updates to a patient’s family member?

Very poor (1), Poor (2), Fair (3), Good (4), Very Good (5)

7. Do you feel this session was helpful in preparing you for intern year?

Yes, No

8. Do you plan to use the information presented here in the future?

Yes, No

9. (Optional) Do you have any recommendations for how this session could be improved?

Free-text short answer
