# Supplementary material for: “Patient's Family Wants an Update”: A Curriculum for Senior Medical Students to Deliver Telephone Updates for Hospitalized Patients
Source: MedEdPORTAL. 2022 May 20;18:11256. doi: 10.15766/mep_2374-8265.11256 (PMC9120304; doi:10.15766/mep_2374-8265.11256)
Supplement: Supplementary file 1 — Family Update Guide.docxFamily Update.pptxPatient Role-Play Cases.docxSelf-Assessment Checklist.docxRetrospective Pre-Post Survey.docx [file mep_2374-8265.11256-s001.zip › A. Family Update Guide.docx]

FAMILY UPDATE GUIDE:

Many patients will want you to provide direct updates to family members or loved ones. Many of these updates are done by telephone, often fitting into the late morning or early afternoon workflow. This template is also effective for in-person conversations.

**Before you start, find a quiet place free of distraction and have the patient’s chart open.**

1. **ASK PATIENT FOR PERMISSION:** Ask patient for permission (or check the chart) to ensure it is okay to provide family members with an update. Clarify who the primary contact for updates should be as well as if there are any limitations on what information can be shared.
2. **INTRODUCTION:**

- Confirm correct contact before sharing private information
  - *“Hi, is this Stephanie?”*
- Introduce yourself and your role on the medical team
  - “*This is Dr. _____ at _____ hospital. I am the general medicine doctor taking care of your father.”*
- Ask permission to have conversation
  - *“Is now an okay time for an update?”*
- Set expectations, particularly if time-constrained
  - “Great, I have about ten minutes to talk today”

1. **CURRENT UNDERSTANDING:**

- Establish baseline of knowledge to avoid duplicating updates and to reveal areas of confusion or misinformation. Remember, family members might receive updates from multiple people (patient, nurse, other doctors).
  - “*I want to understand how much you already know about your family member’s condition. What is the latest update that you received?”*

1. **OVERALL ASSESSMENT:**

- Summarize overall condition of patient and trajectory.
  - *“Your father’s condition continues to improve and he no longer needs to be in the intensive care unit. He still needs some extra oxygen but is feeling and looking much better. We will be moving him to a new bed in the regular part of the hospital soon.”*
  - “I’m afraid I have some serious news. [Pause.] Your father has needed more and more oxygen over the past several hours and so he needs to move to the intensive care unit. I cannot predict exactly what will happen next, but I worry that he might need to go on a breathing machine with a breathing tube soon.”

1. **INTERVAL EVENTS:**

- Describe other significant interval events. This would include things like major events: falls, new delirium, new diagnoses. Exclude insignificant details (such as minor lab changes, minor medication dose changes).

1. **FOCUSED UPDATES:**

- Provide updates for each major medical problem, one at a time.
  - *“We are treating three main problems: pneumonia, his abnormal heart rhythm, and his nighttime delirium. Let’s start with the pneumonia…”*
- Remember that this is also your opportunity to gather more information about the patient from somebody who knows them well:
  - *“Last night, he became confused for a few hours and wasn’t sure where he was. Has this happened during his previous hospitalizations, or does this ever happen at home?”*

1. **PLAN:**

- Outline plan of care for each major medical problem. For complex patients, you can discuss updates and plan for each individual medical problem before moving on to the next problem.
  - *“For his pneumonia, we are going to continue antibiotics through his vein and supporting him with extra oxygen, but if he continues to improve, we might be able to switch to antibiotic pills as soon as tomorrow.*
- When appropriate, outline disposition plan and what criteria need to be met for discharge. Communication of discharge planning with family in advance will help for smoother transitions.
  - *“My best estimate is that he will need to stay in the hospital for another two or three days. We would like to see that he no longer needs extra oxygen first and that he is still getting better after the switch to pill antibiotics.”*

1. **CONFIRM UNDERSTANDING:**

- If long or complex conversation, give quick recap of conversation
- Ensure understanding of new information
  - (If complex problem) “*I know this was a lot of new information. Could I have you repeat it back to make sure I explained it well?”*
- Open dialogue for questions
  - “What other questions do you have?” or “What else can I clarify?”
  - Responses range from no questions to an excessively long list of questions. Expectations have already been set that there are time restraints on the update. If questions are starting to become inappropriately detail-oriented or too numerous, it is okay to set boundaries and gently reinforce time limits. *“I’m sorry Stephanie, I only have another minute to talk as I have to go see other patients. Even though we might not go over all the details on this call, I assure you our team is being thorough. I understand these details are important and I am happy to call you back tomorrow to talk through more of the details with you.”*

1. **CONCLUSION**

- Say goodbye and set clear expectation for when next update will be. If a patient is very sick, emphasize that you will call sooner if anything changes.
  - *“It was nice speaking with you today. Since he is moving out of the ICU, a different team of doctors will soon be taking care of him. I will let them know that we spoke and you can expect regular updates from them as well.”*

**IMPORTANT CONSIDERATIONS:**

- Be cognizant of level of medical literacy and limit use of jargon
- Express empathy where appropriate; these updates are often emotionally challenging and establishing a human connection is important
- Be honest with your updates even if the news is bad; it is also important to acknowledge when you do not know an answer to a question
- Family members will sometimes ask too much of you (multiple updates per day, excessive list of questions, special exceptions regarding visitor policies, etc.). It is important to set and enforce boundaries to protect your own time and hospital policies.
- Ensure families know to contact medical team (calling operator, unit, RN, etc.)
- It can be very helpful to document a conversation in the chart (even if a brief note), especially if a family is unhappy with any aspect of their care. Phrases such as “[Name of family member] expressed understanding and all questions were answered” are particularly helpful in certain circumstances.
