# Supplementary material for: “Patient's Family Wants an Update”: A Curriculum for Senior Medical Students to Deliver Telephone Updates for Hospitalized Patients
Source: MedEdPORTAL. 2022 May 20;18:11256. doi: 10.15766/mep_2374-8265.11256 (PMC9120304; doi:10.15766/mep_2374-8265.11256)
Supplement: Supplementary file 1 — Family Update Guide.docxFamily Update.pptxPatient Role-Play Cases.docxSelf-Assessment Checklist.docxRetrospective Pre-Post Survey.docx [file mep_2374-8265.11256-s001.zip › B. Family Update.pptx]

## Slide 1
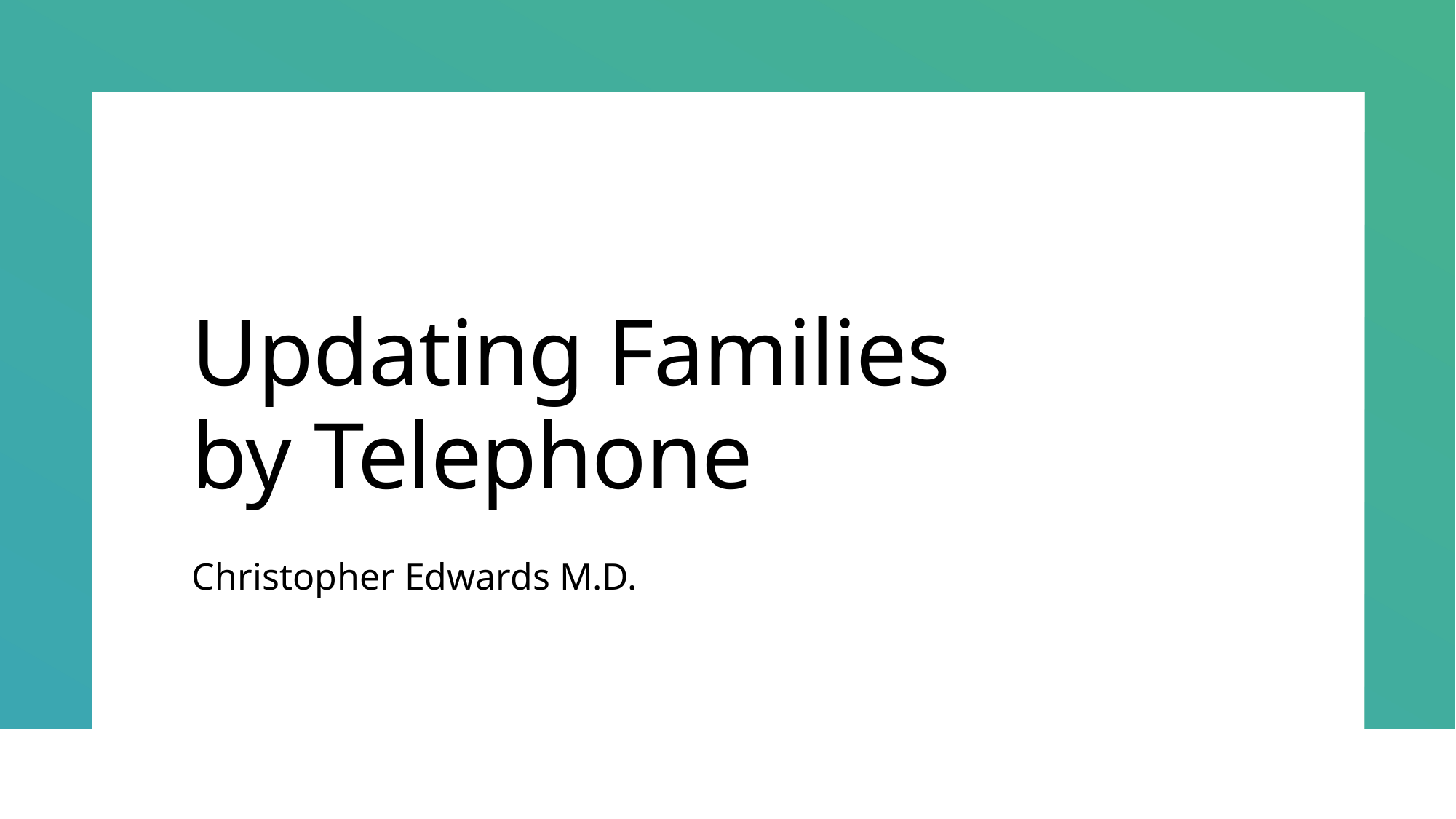

# Updating Families by Telephone
Christopher Edwards M.D.

## Slide 2
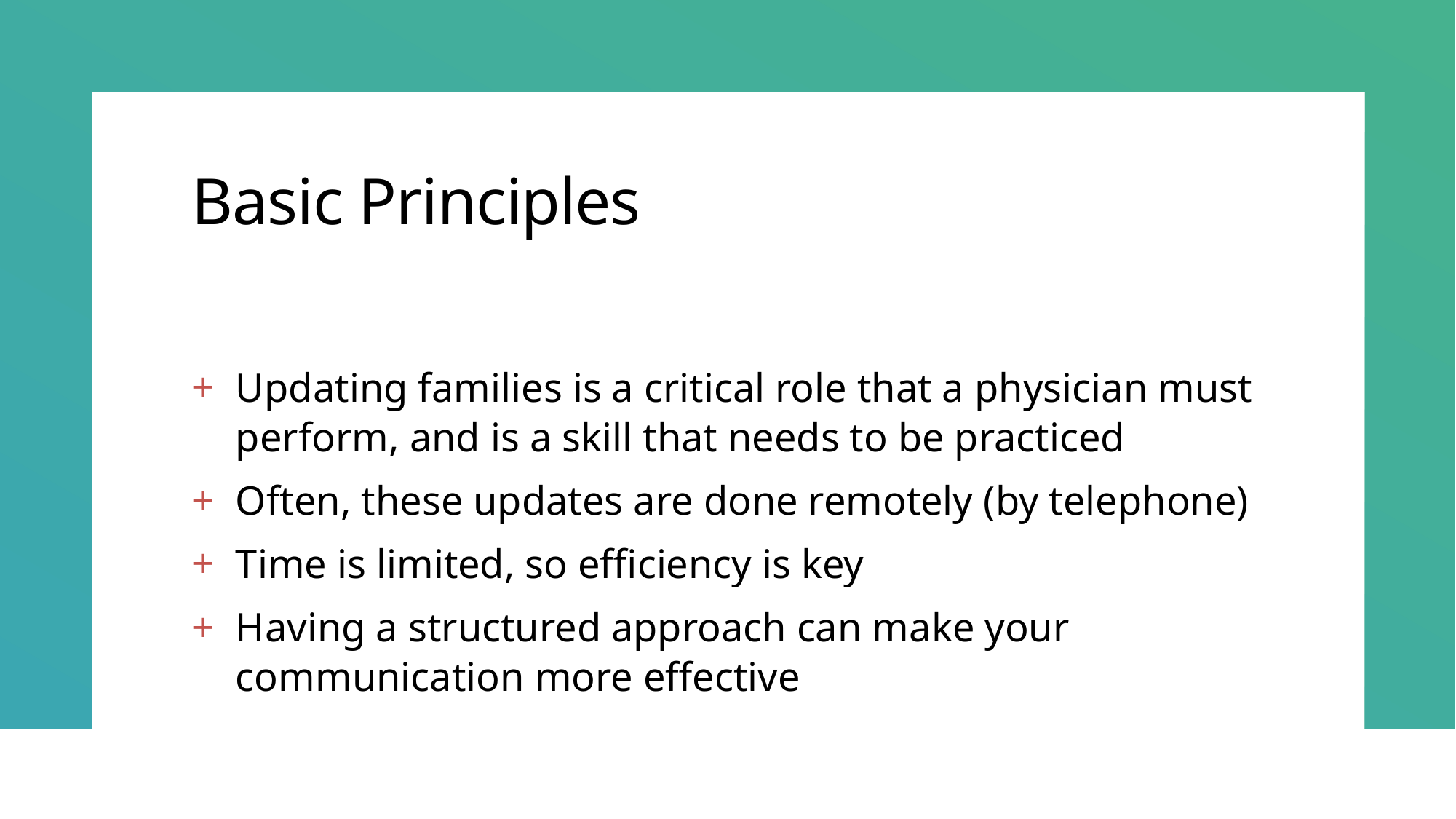

# Basic Principles
Updating families is a critical role that a physician must perform, and is a skill that needs to be practiced
Often, these updates are done remotely (by telephone)
Time is limited, so efficiency is key
Having a structured approach can make your communication more effective

## Slide 3
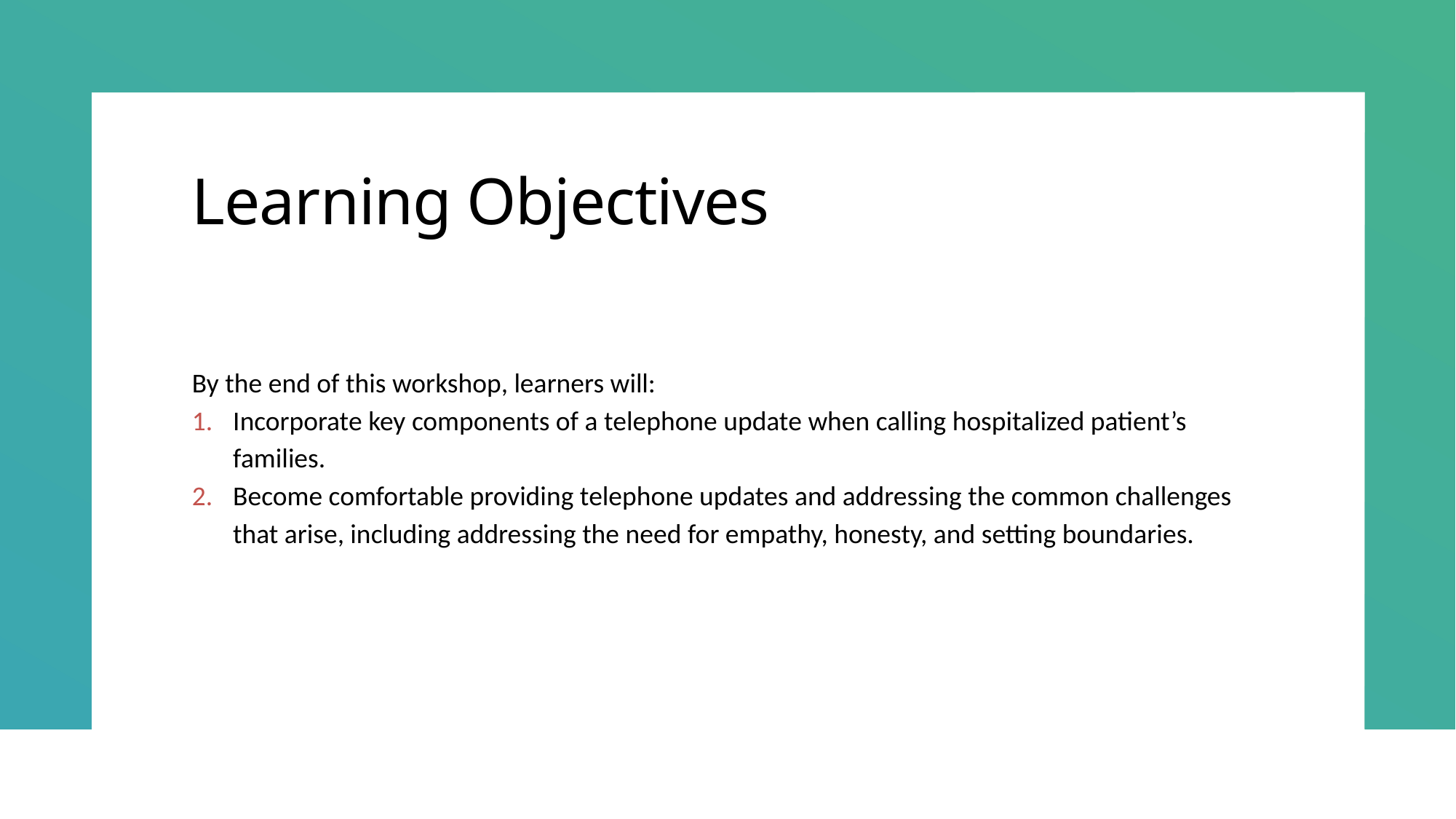

# Learning Objectives
By the end of this workshop, learners will:
Incorporate key components of a telephone update when calling hospitalized patient’s families.
Become comfortable providing telephone updates and addressing the common challenges that arise, including addressing the need for empathy, honesty, and setting boundaries.

## Slide 4
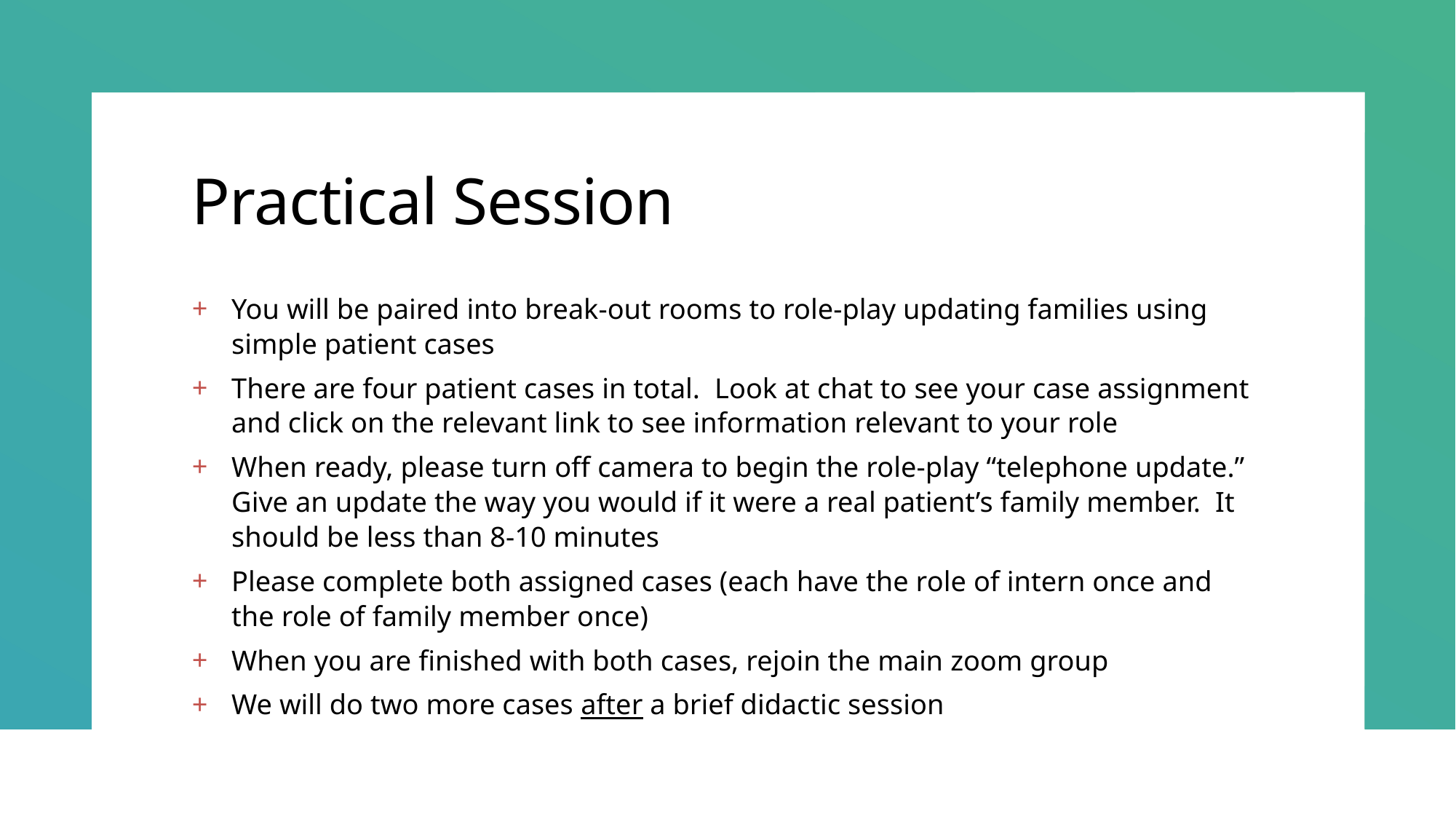

# Practical Session
You will be paired into break-out rooms to role-play updating families using simple patient cases
There are four patient cases in total. Look at chat to see your case assignment and click on the relevant link to see information relevant to your role
When ready, please turn off camera to begin the role-play “telephone update.” Give an update the way you would if it were a real patient’s family member. It should be less than 8-10 minutes
Please complete both assigned cases (each have the role of intern once and the role of family member once)
When you are finished with both cases, rejoin the main zoom group
We will do two more cases after a brief didactic session

## Slide 5
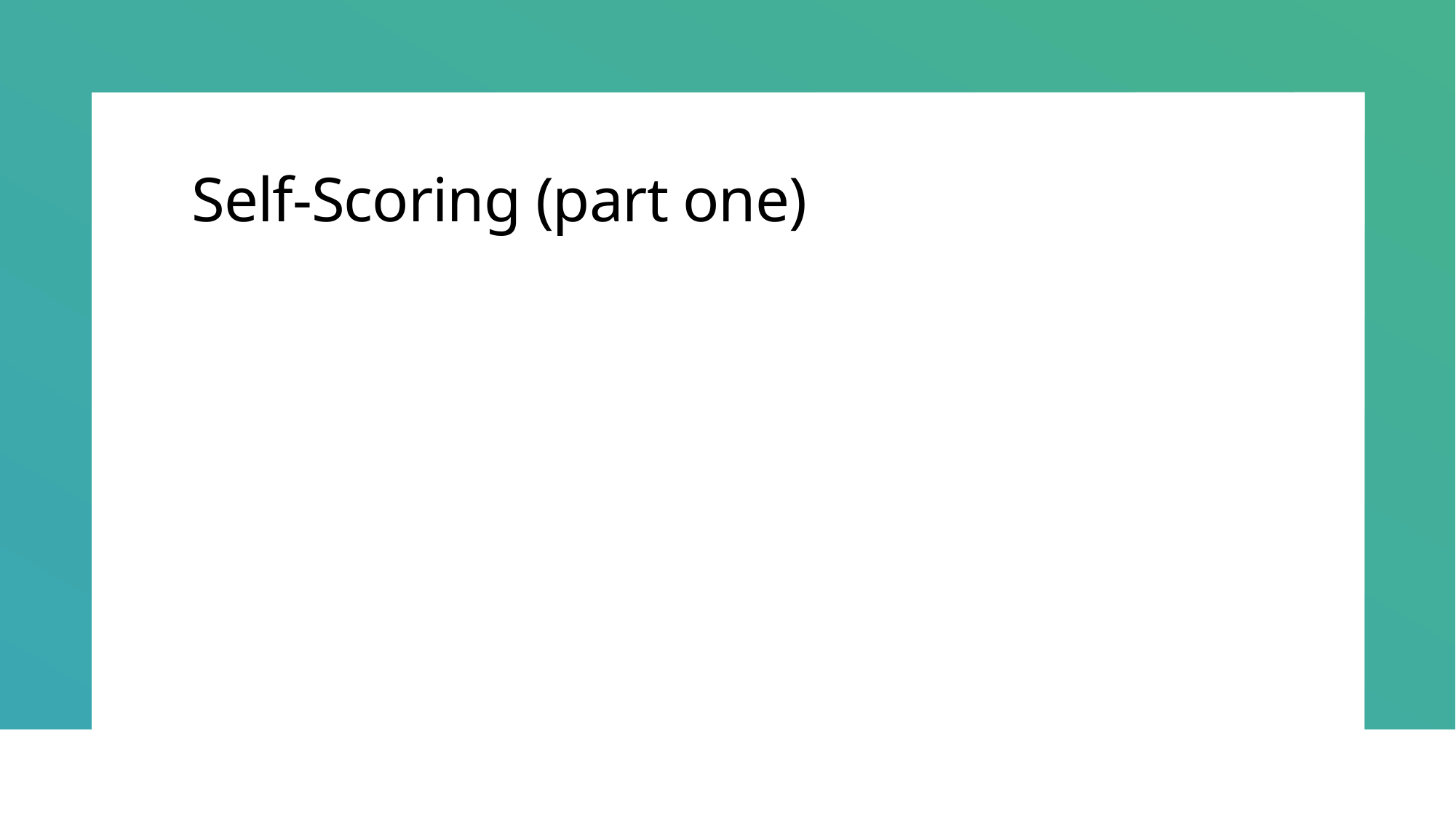

# Self-Scoring (part one)

## Slide 6
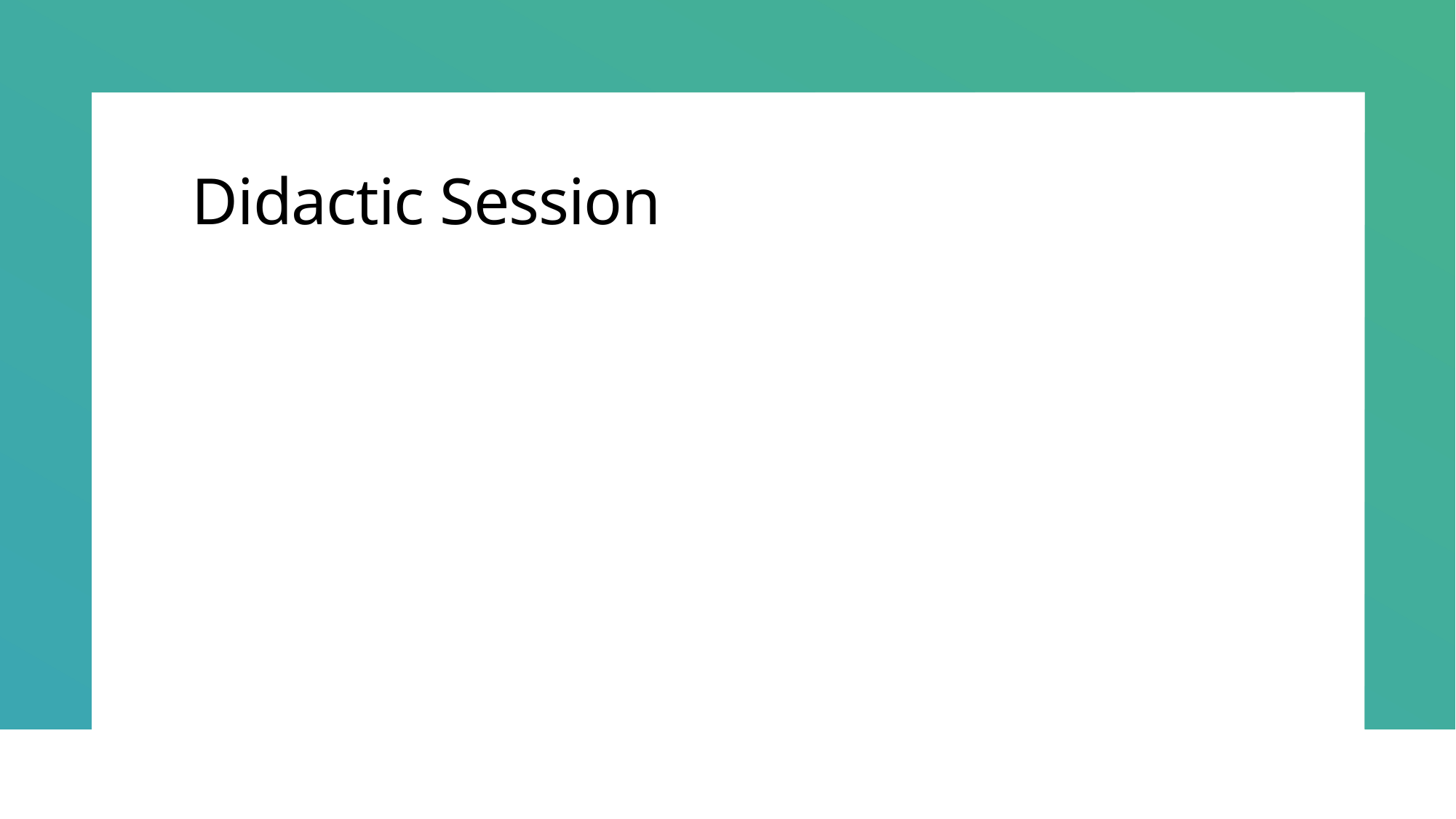

# Didactic Session

## Slide 7
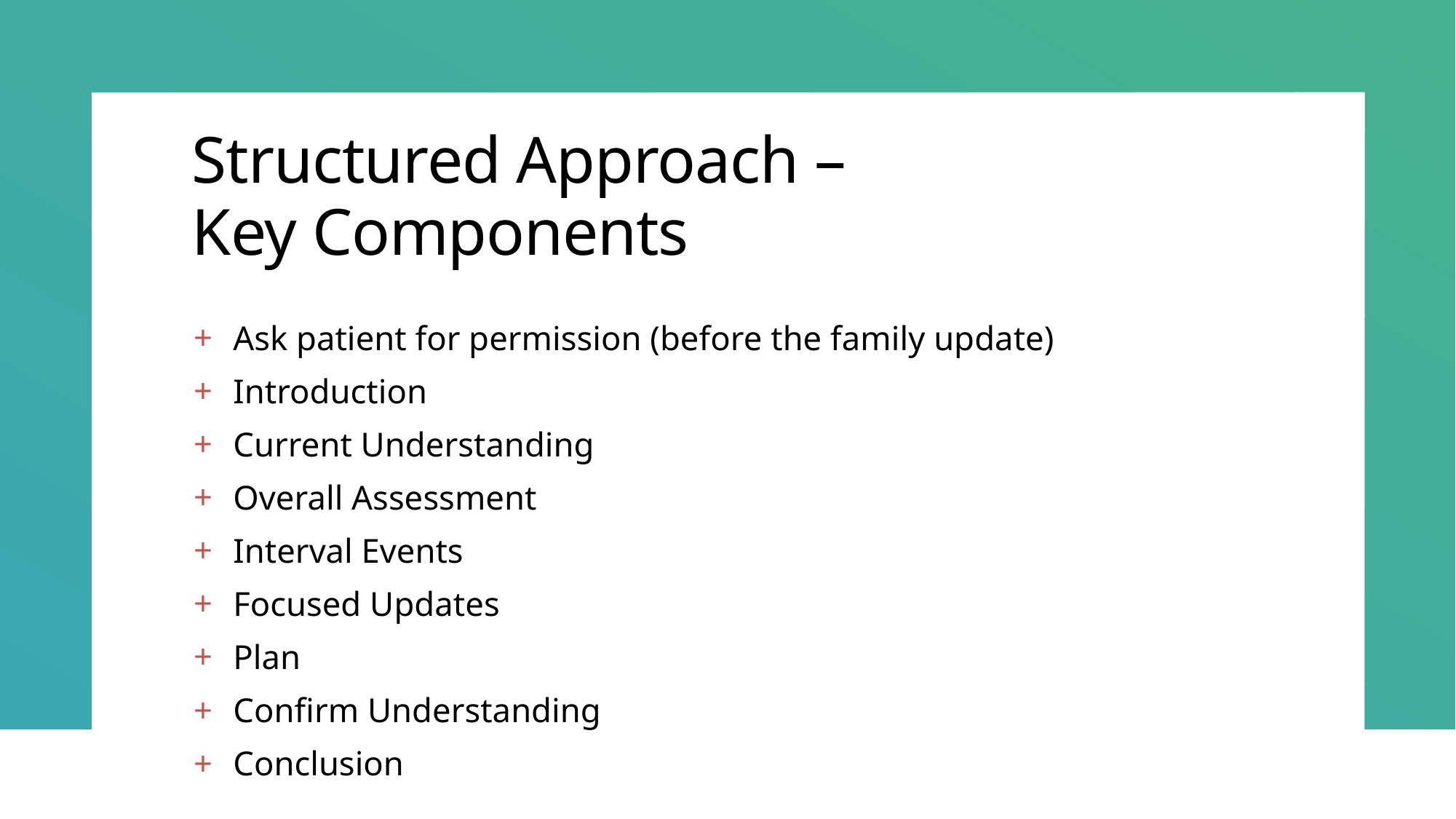

# Structured Approach – Key Components
Ask patient for permission (before the family update)
Introduction
Current Understanding
Overall Assessment
Interval Events
Focused Updates
Plan
Confirm Understanding
Conclusion

## Slide 8
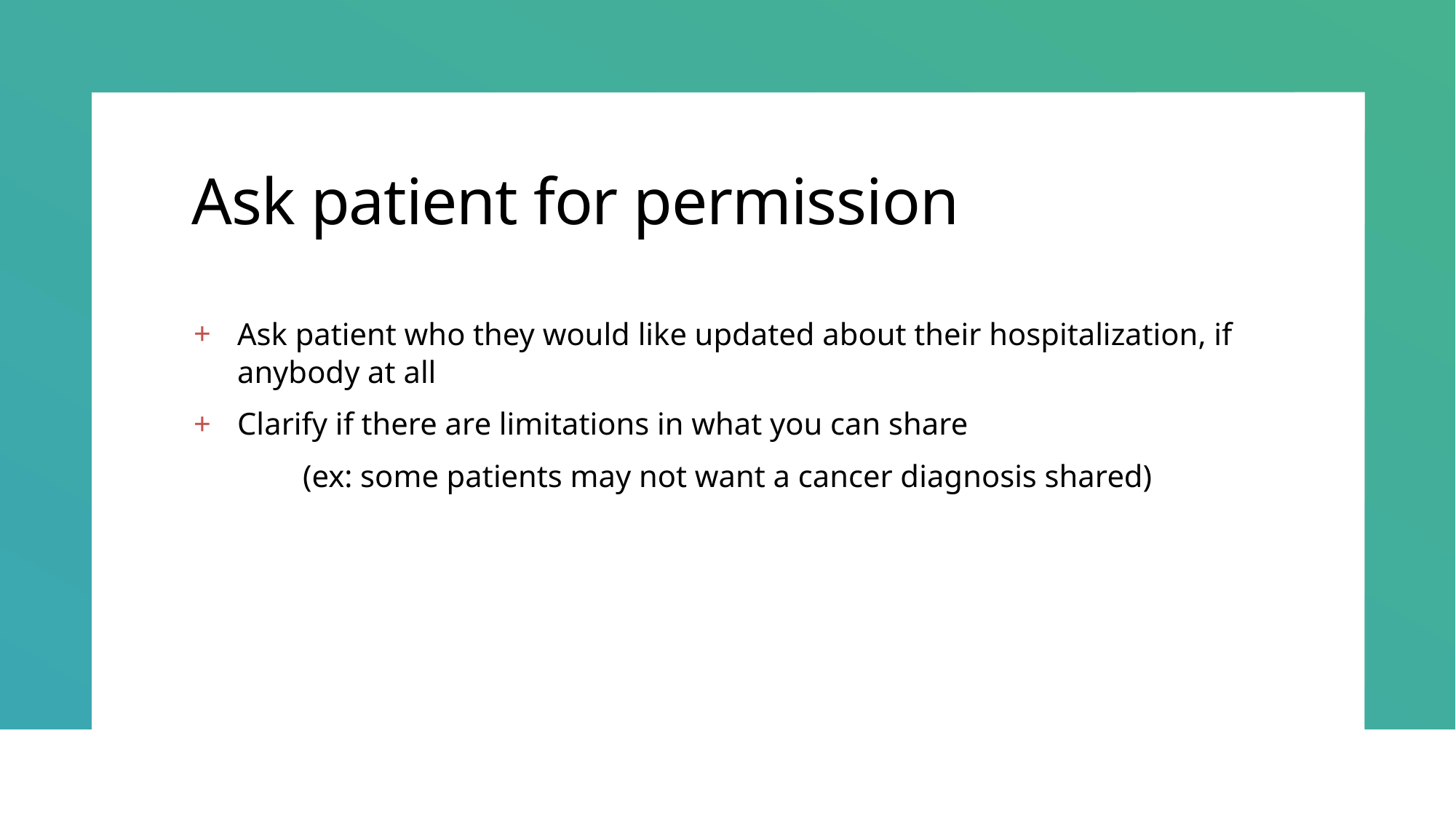

# Ask patient for permission
Ask patient who they would like updated about their hospitalization, if anybody at all
Clarify if there are limitations in what you can share
	(ex: some patients may not want a cancer diagnosis shared)

## Slide 9
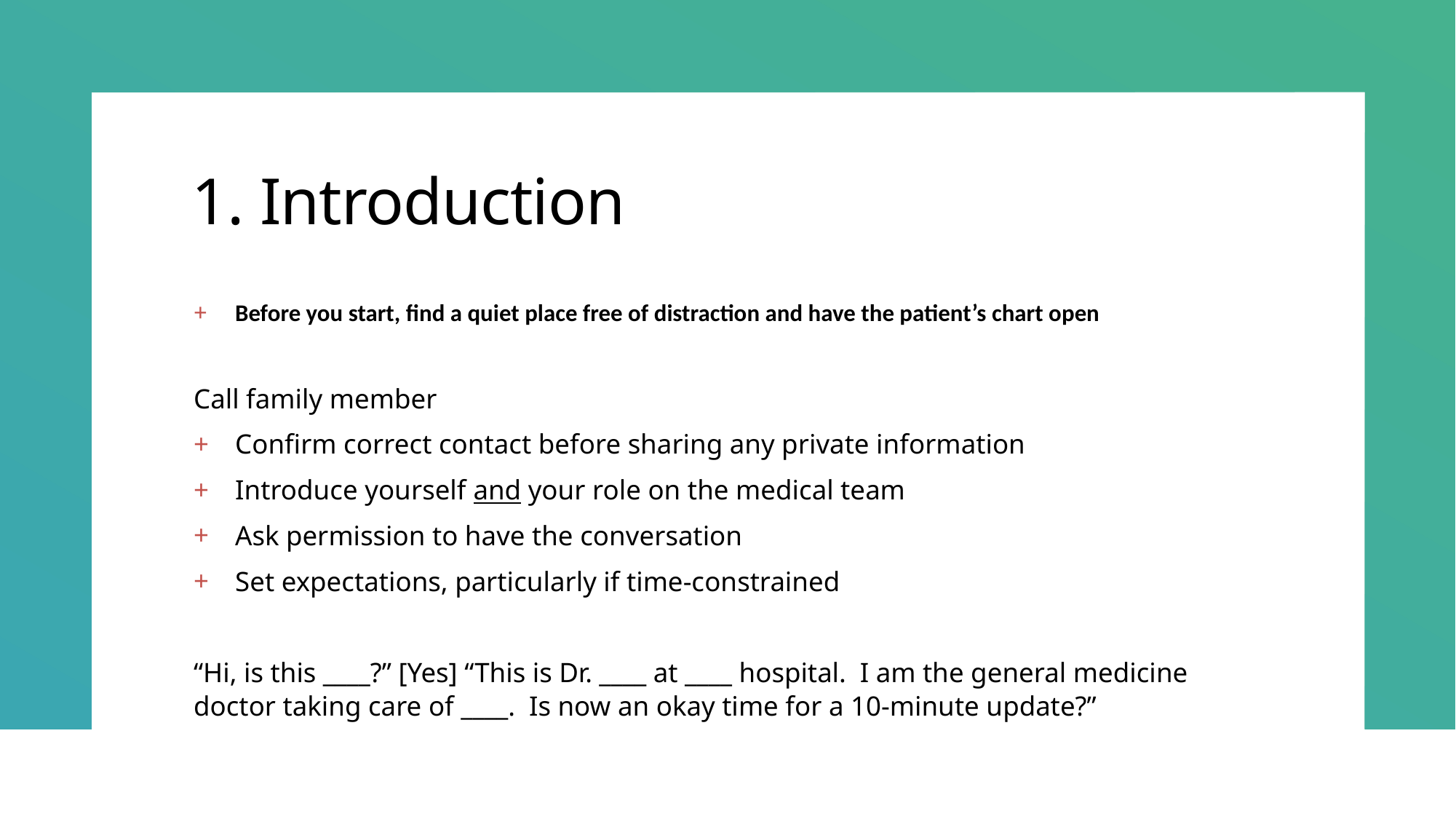

# 1. Introduction
Before you start, find a quiet place free of distraction and have the patient’s chart open
Call family member
Confirm correct contact before sharing any private information
Introduce yourself and your role on the medical team
Ask permission to have the conversation
Set expectations, particularly if time-constrained
“Hi, is this ____?” [Yes] “This is Dr. ____ at ____ hospital. I am the general medicine doctor taking care of ____. Is now an okay time for a 10-minute update?”

## Slide 10
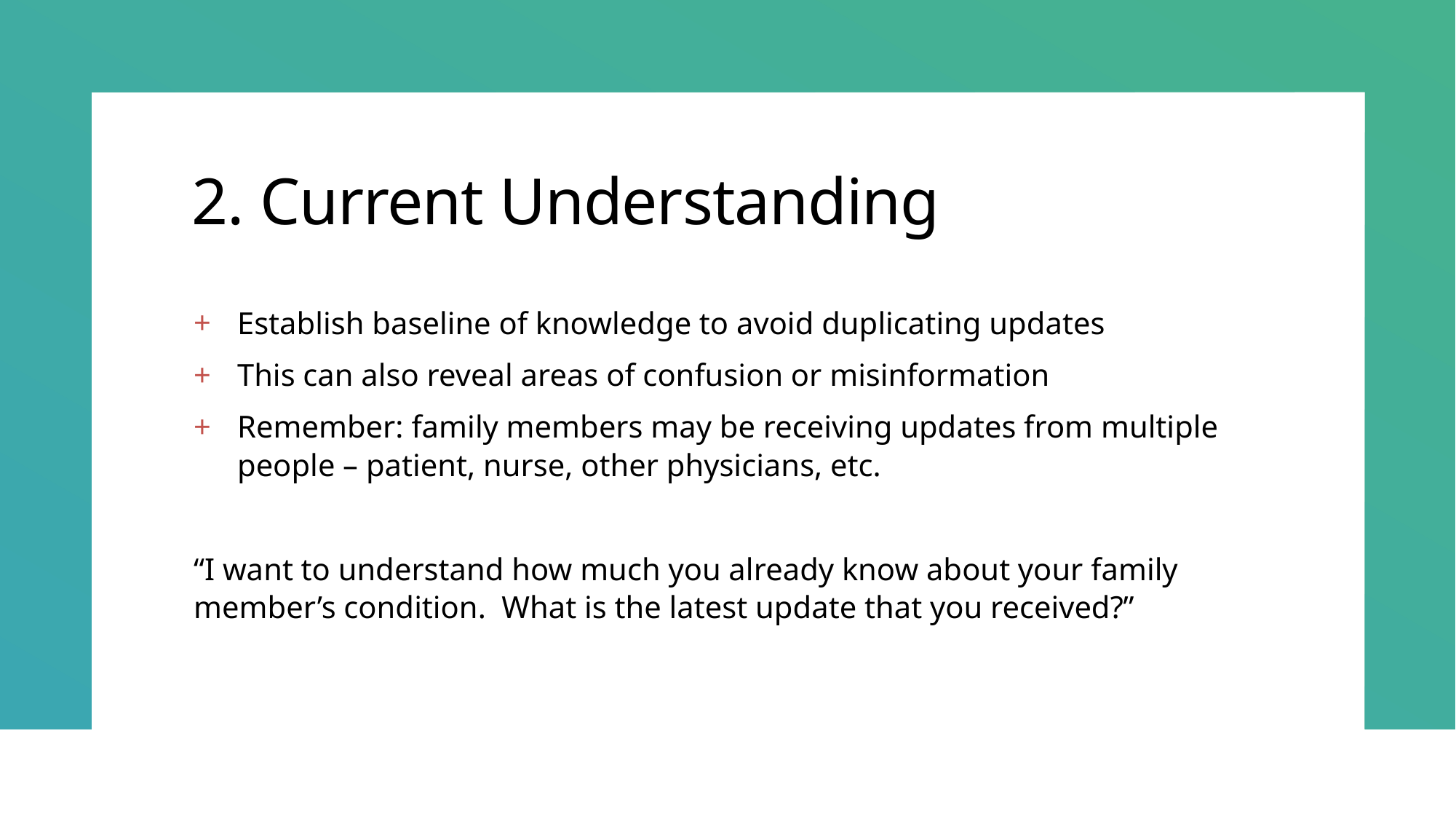

# 2. Current Understanding
Establish baseline of knowledge to avoid duplicating updates
This can also reveal areas of confusion or misinformation
Remember: family members may be receiving updates from multiple people – patient, nurse, other physicians, etc.
“I want to understand how much you already know about your family member’s condition. What is the latest update that you received?”

## Slide 11
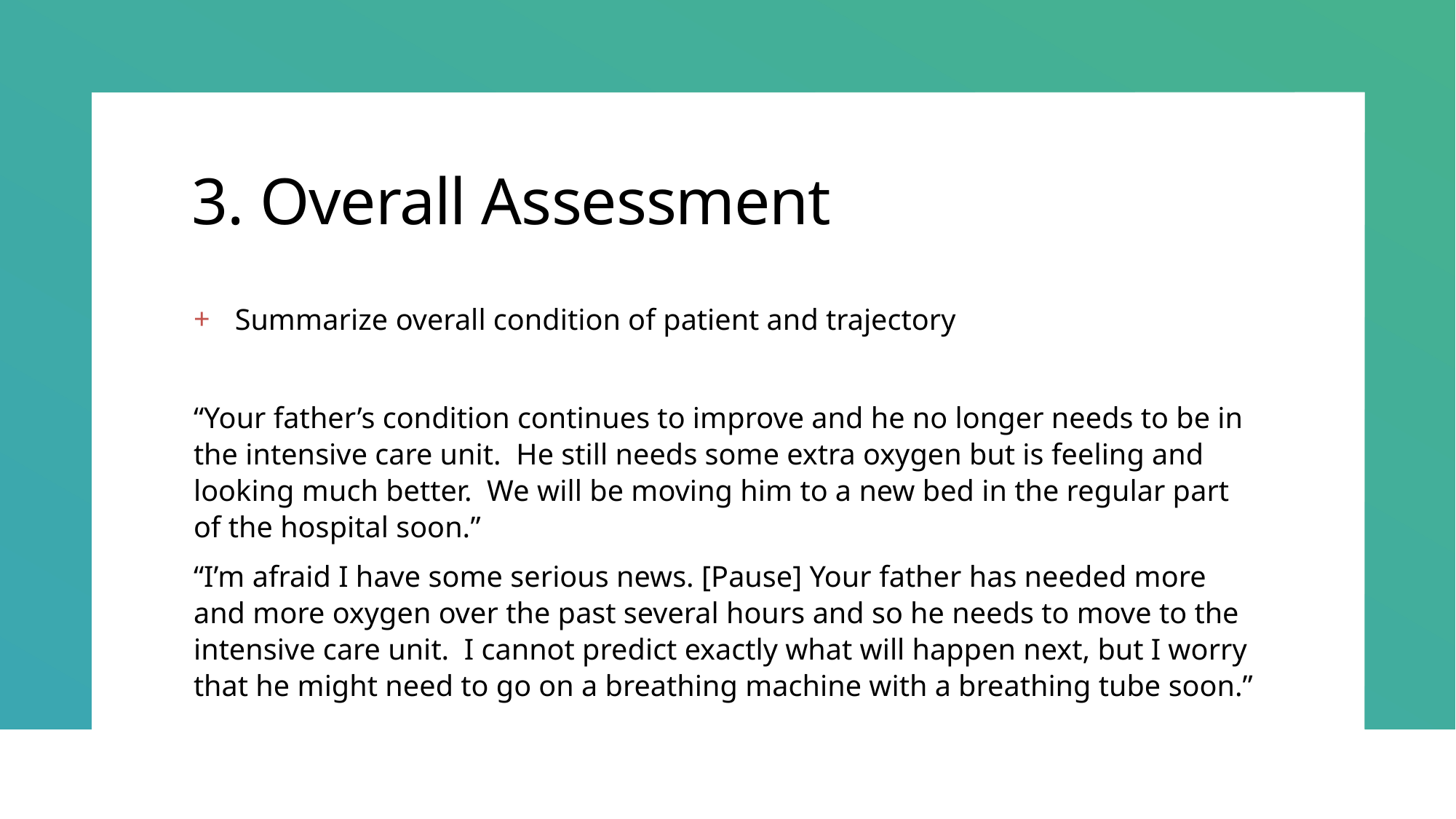

# 3. Overall Assessment
Summarize overall condition of patient and trajectory
“Your father’s condition continues to improve and he no longer needs to be in the intensive care unit. He still needs some extra oxygen but is feeling and looking much better. We will be moving him to a new bed in the regular part of the hospital soon.”
“I’m afraid I have some serious news. [Pause] Your father has needed more and more oxygen over the past several hours and so he needs to move to the intensive care unit. I cannot predict exactly what will happen next, but I worry that he might need to go on a breathing machine with a breathing tube soon.”

## Slide 12
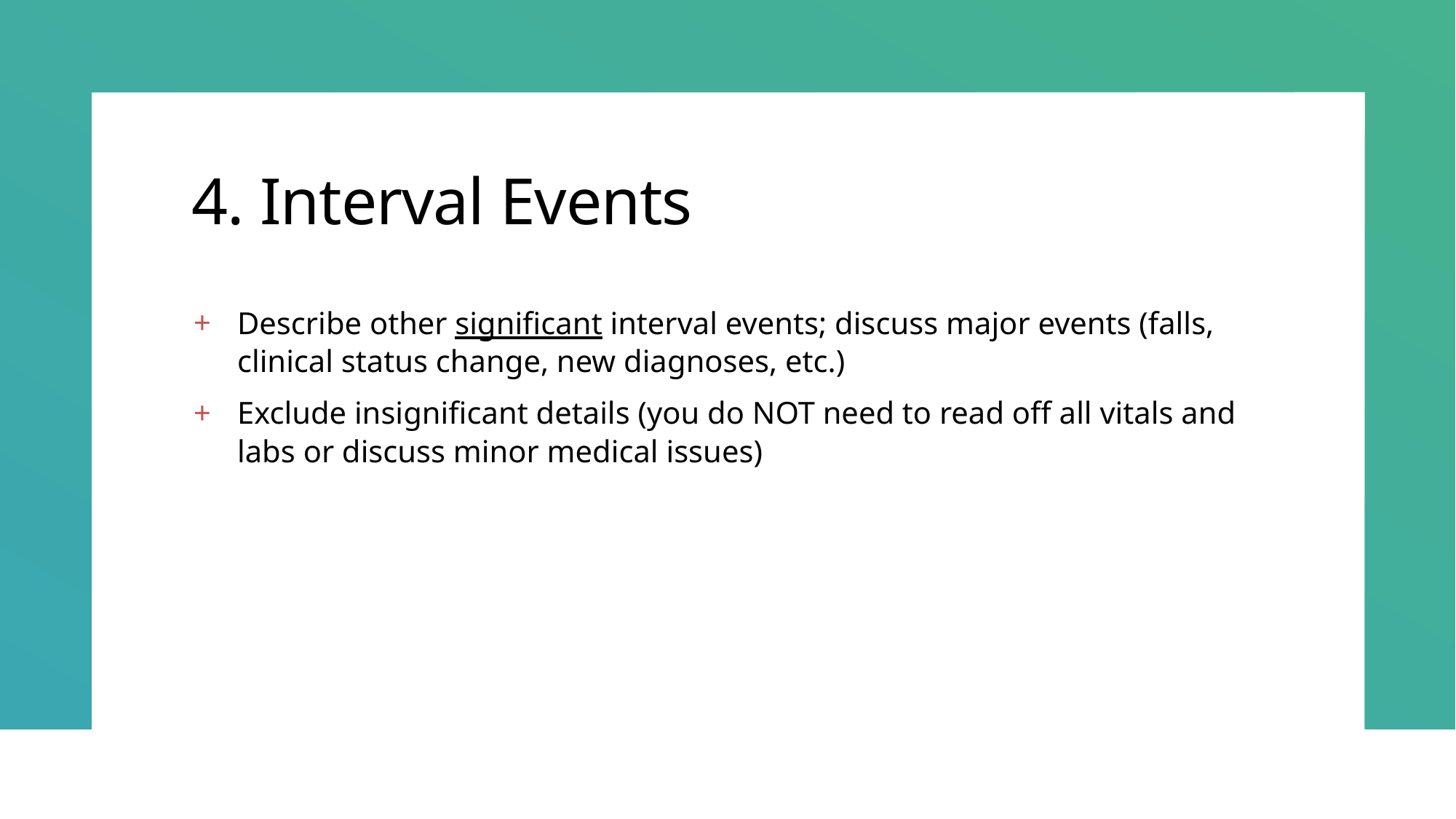

# 4. Interval Events
Describe other significant interval events; discuss major events (falls, clinical status change, new diagnoses, etc.)
Exclude insignificant details (you do NOT need to read off all vitals and labs or discuss minor medical issues)

## Slide 13
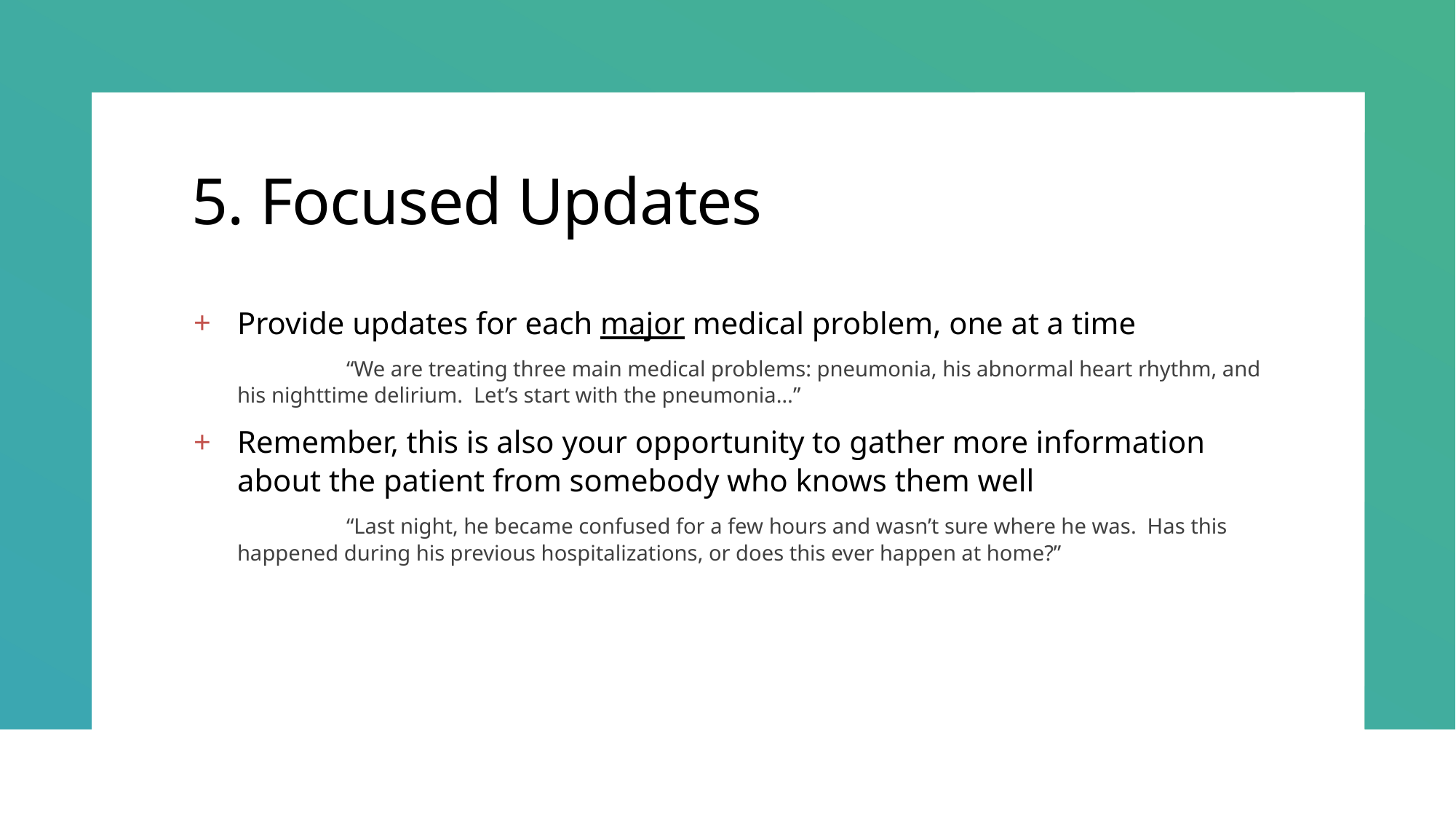

# 5. Focused Updates
Provide updates for each major medical problem, one at a time
	“We are treating three main medical problems: pneumonia, his abnormal heart rhythm, and his nighttime delirium. Let’s start with the pneumonia…”
Remember, this is also your opportunity to gather more information about the patient from somebody who knows them well
	“Last night, he became confused for a few hours and wasn’t sure where he was. Has this happened during his previous hospitalizations, or does this ever happen at home?”

## Slide 14
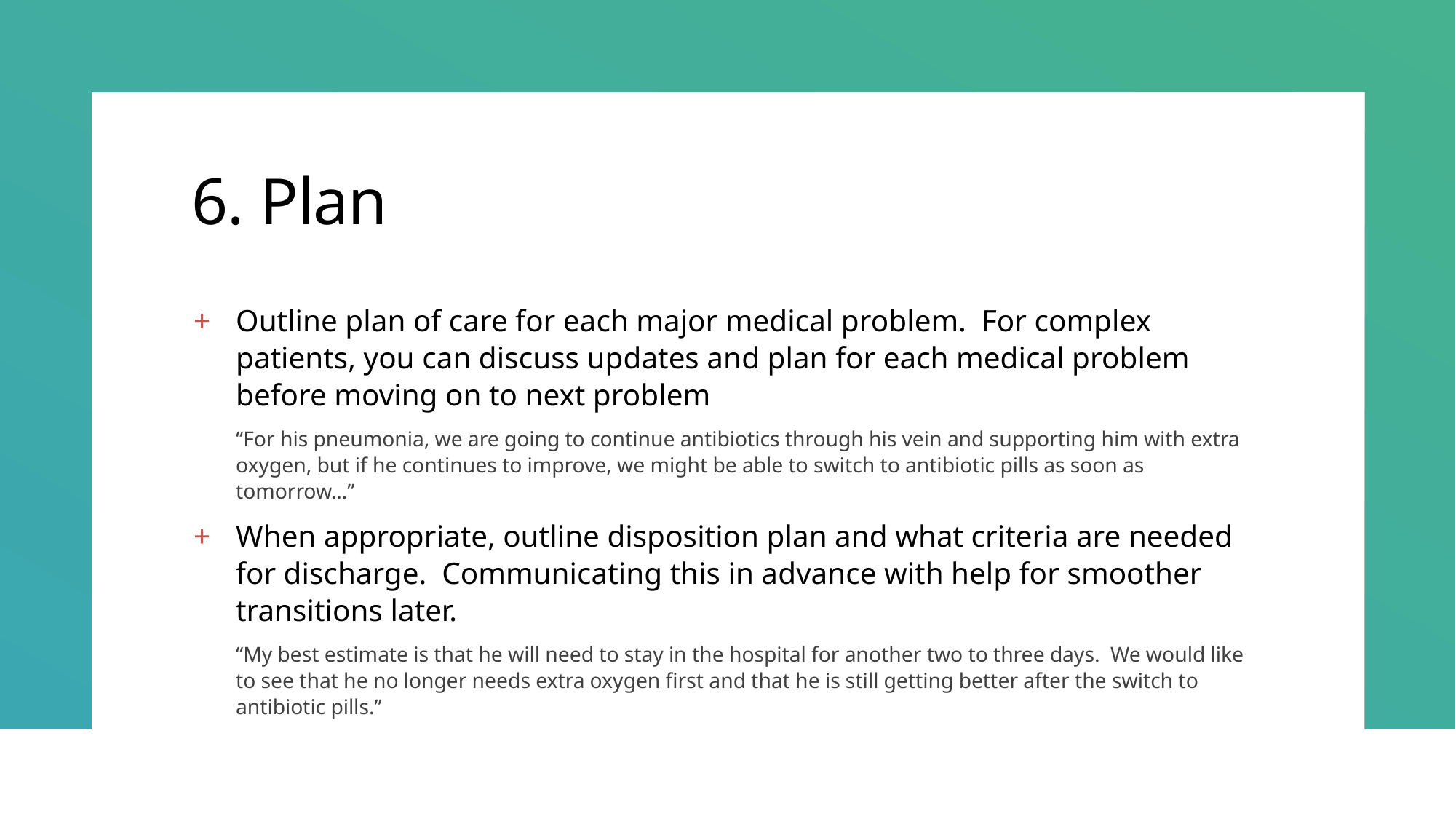

# 6. Plan
Outline plan of care for each major medical problem. For complex patients, you can discuss updates and plan for each medical problem before moving on to next problem
	“For his pneumonia, we are going to continue antibiotics through his vein and supporting him with extra oxygen, but if he continues to improve, we might be able to switch to antibiotic pills as soon as tomorrow…”
When appropriate, outline disposition plan and what criteria are needed for discharge. Communicating this in advance with help for smoother transitions later.
	“My best estimate is that he will need to stay in the hospital for another two to three days. We would like to see that he no longer needs extra oxygen first and that he is still getting better after the switch to antibiotic pills.”

## Slide 15
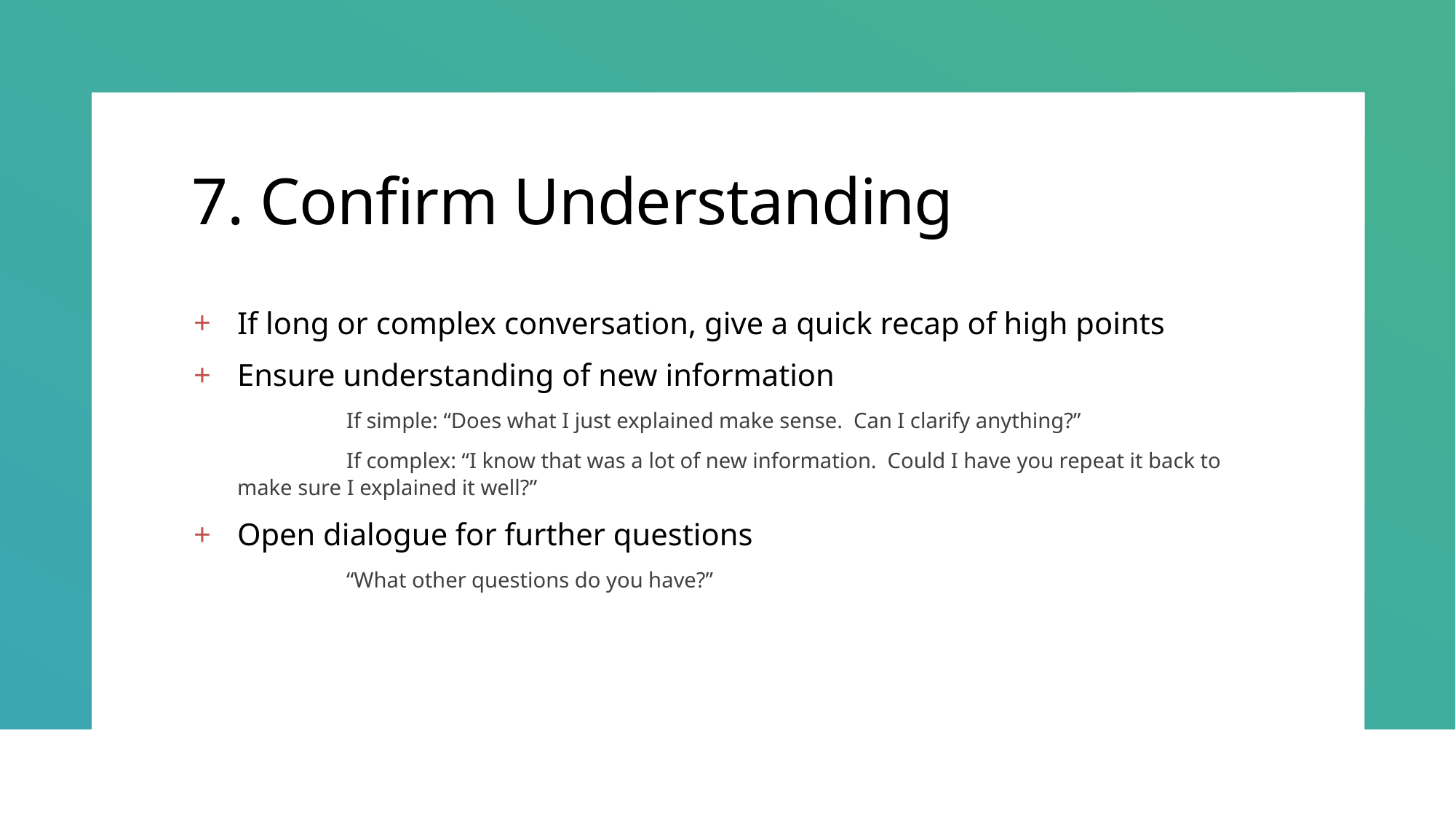

# 7. Confirm Understanding
If long or complex conversation, give a quick recap of high points
Ensure understanding of new information
	If simple: “Does what I just explained make sense. Can I clarify anything?”
	If complex: “I know that was a lot of new information. Could I have you repeat it back to make sure I explained it well?”
Open dialogue for further questions
	“What other questions do you have?”

## Slide 16
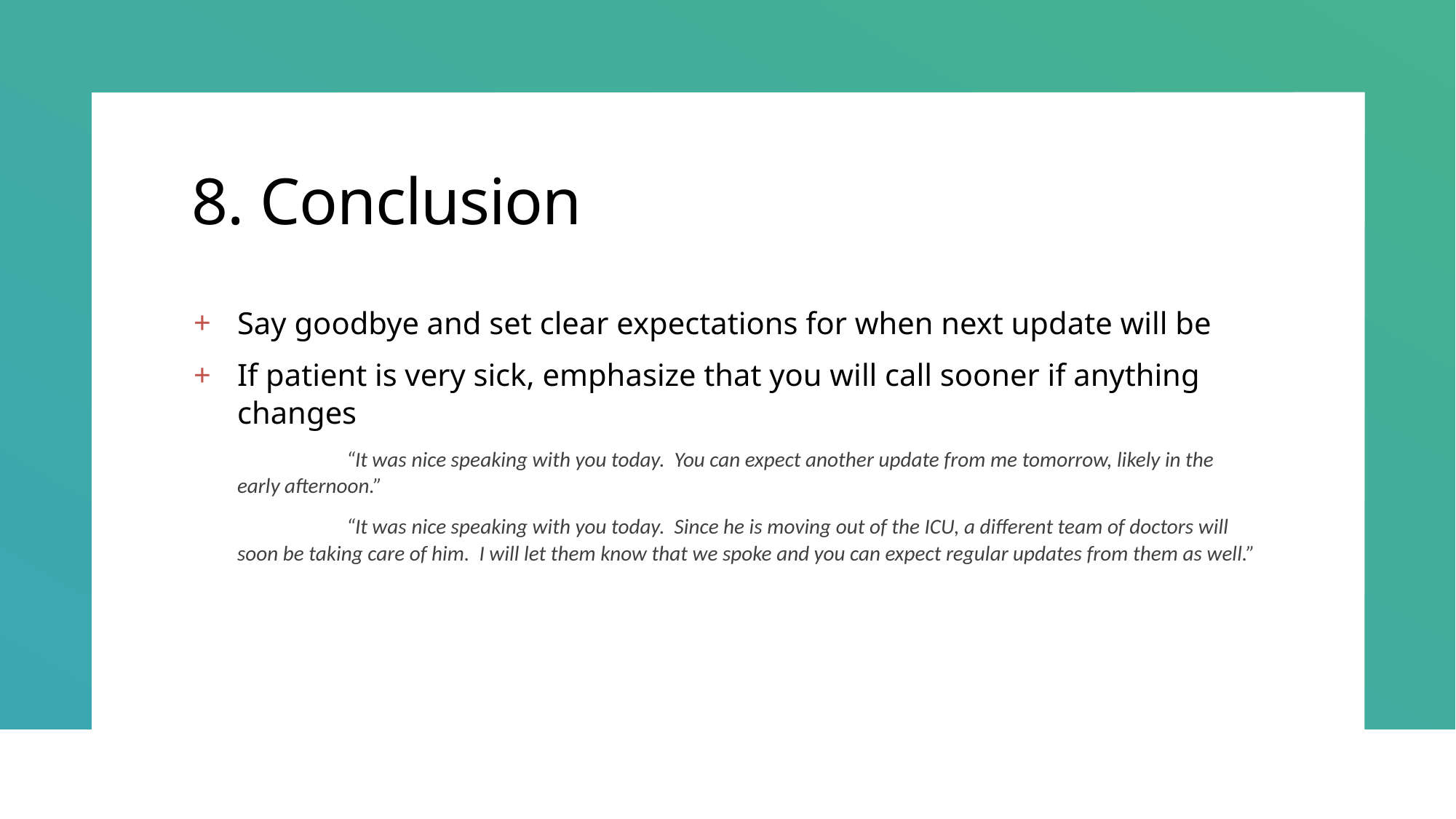

# 8. Conclusion
Say goodbye and set clear expectations for when next update will be
If patient is very sick, emphasize that you will call sooner if anything changes
	“It was nice speaking with you today. You can expect another update from me tomorrow, likely in the early afternoon.”
	“It was nice speaking with you today. Since he is moving out of the ICU, a different team of doctors will soon be taking care of him. I will let them know that we spoke and you can expect regular updates from them as well.”

## Slide 17
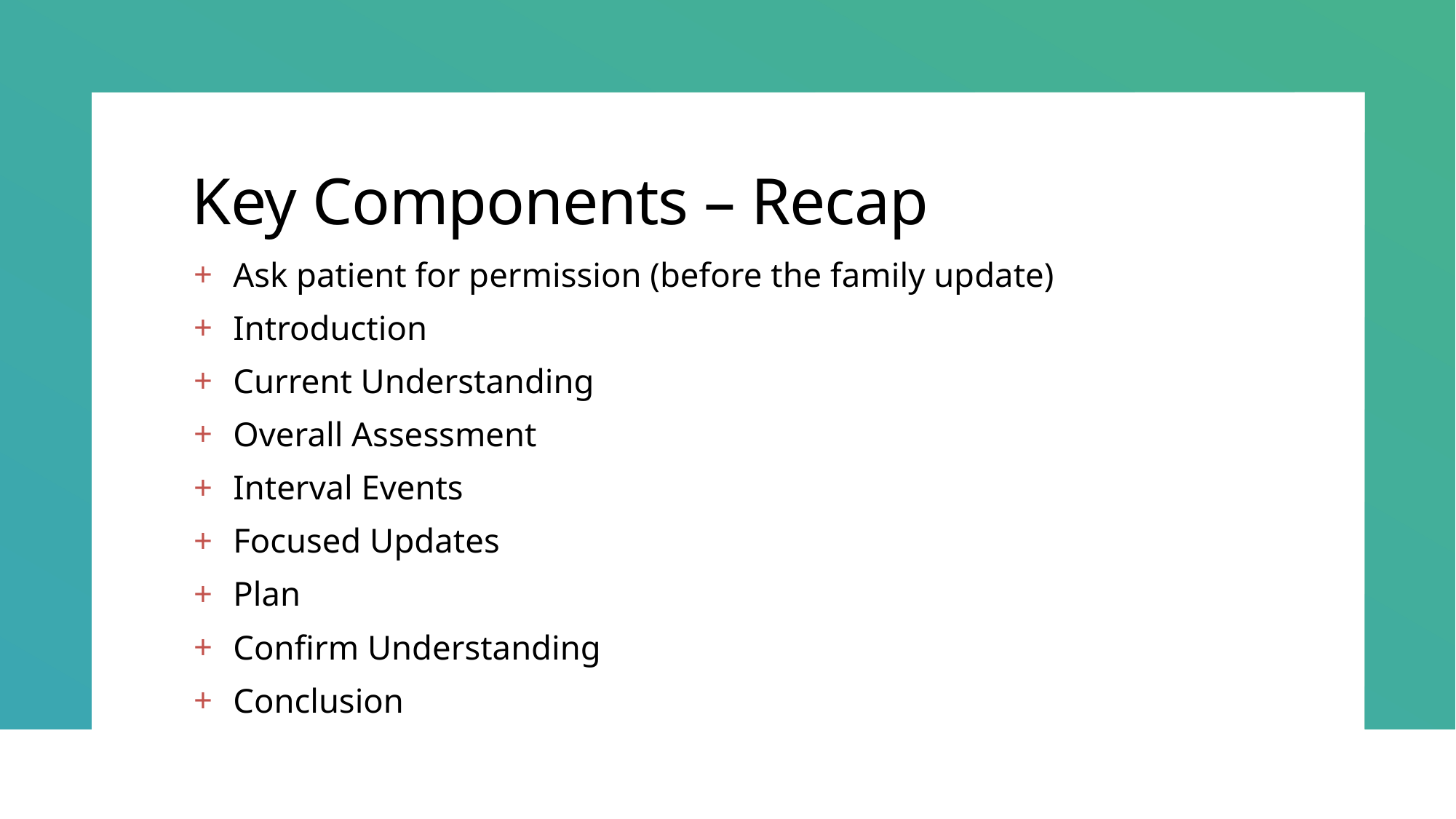

# Key Components – Recap
Ask patient for permission (before the family update)
Introduction
Current Understanding
Overall Assessment
Interval Events
Focused Updates
Plan
Confirm Understanding
Conclusion

## Slide 18
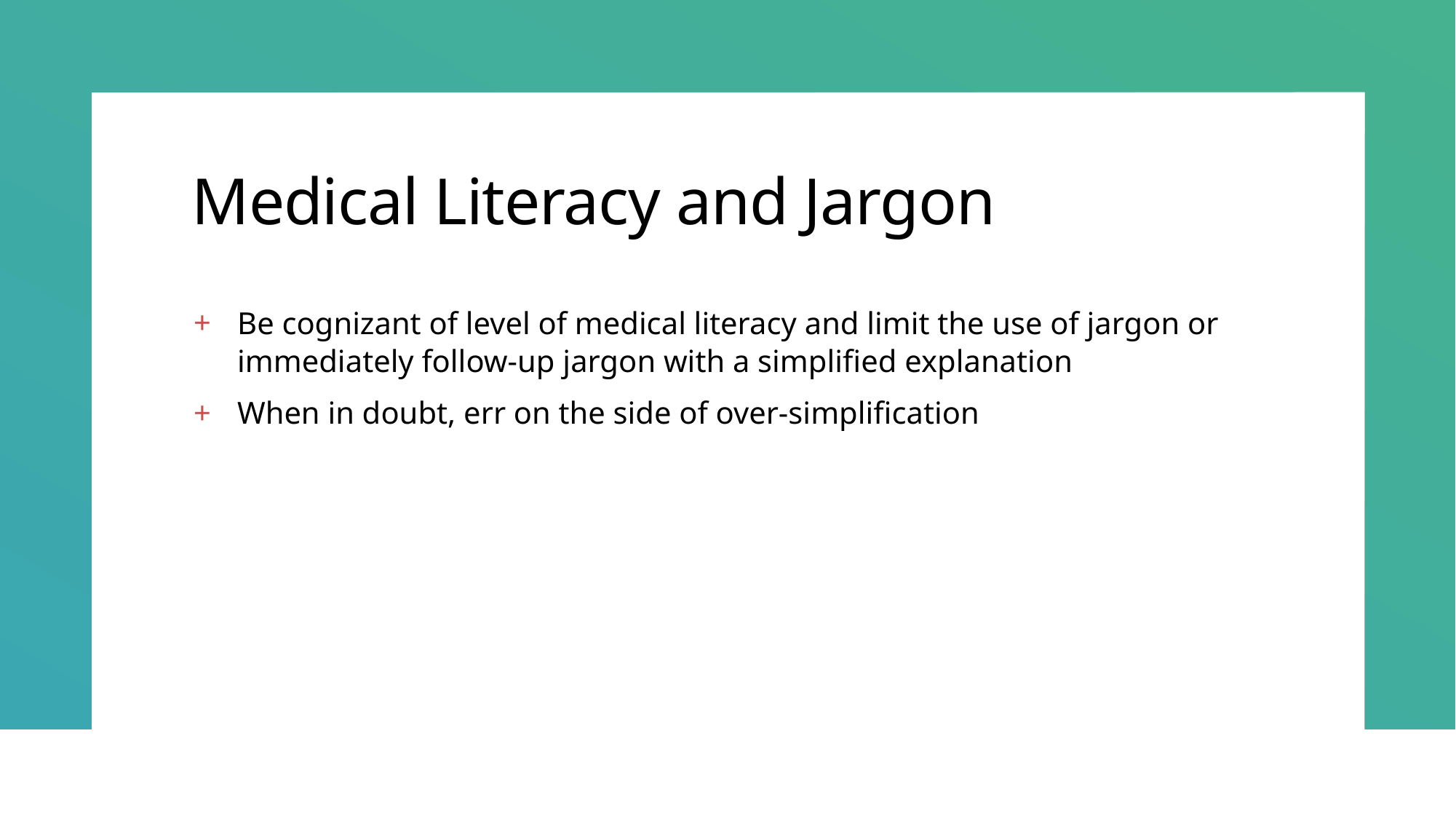

# Medical Literacy and Jargon
Be cognizant of level of medical literacy and limit the use of jargon or immediately follow-up jargon with a simplified explanation
When in doubt, err on the side of over-simplification

## Slide 19
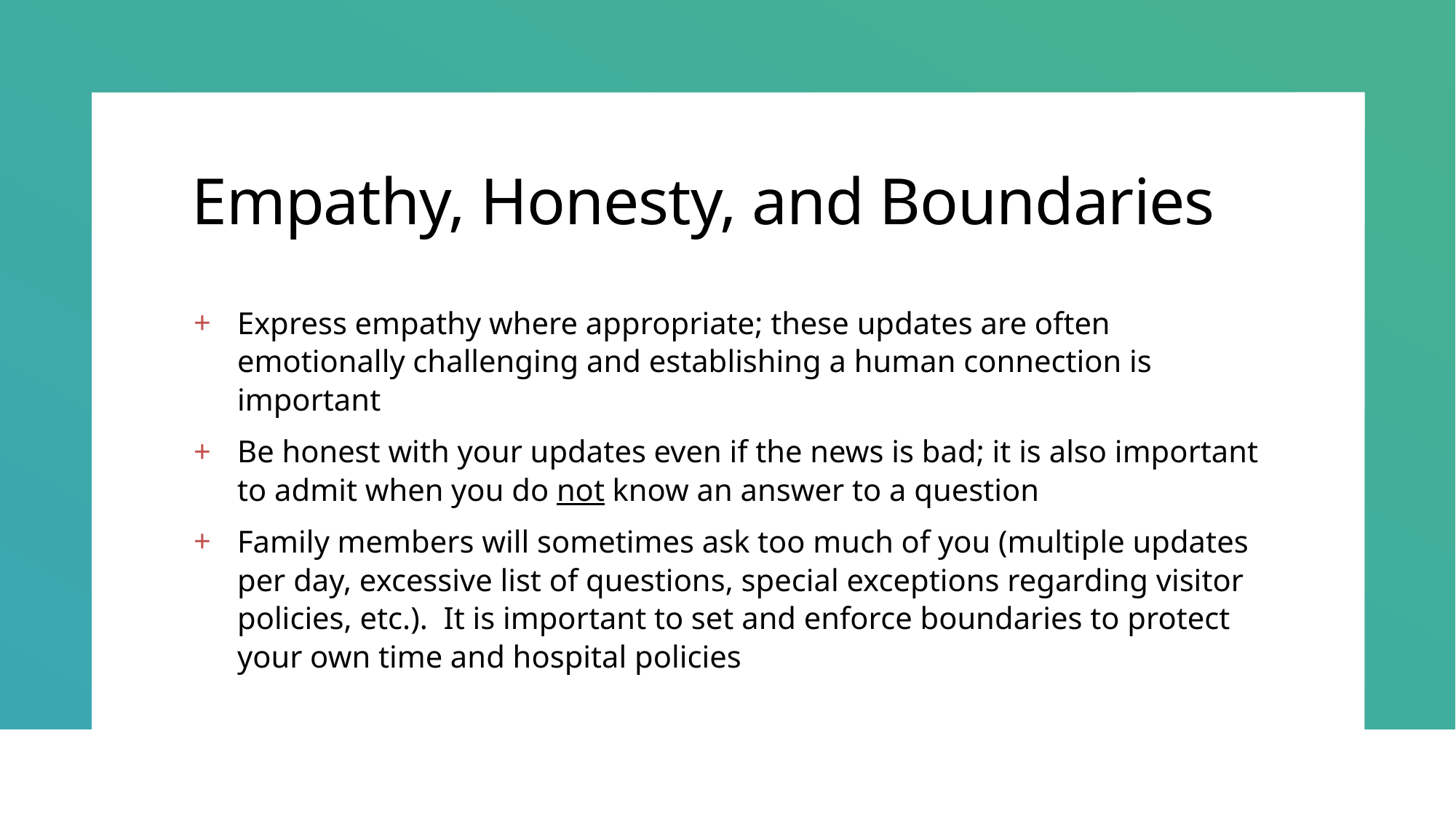

# Empathy, Honesty, and Boundaries
Express empathy where appropriate; these updates are often emotionally challenging and establishing a human connection is important
Be honest with your updates even if the news is bad; it is also important to admit when you do not know an answer to a question
Family members will sometimes ask too much of you (multiple updates per day, excessive list of questions, special exceptions regarding visitor policies, etc.). It is important to set and enforce boundaries to protect your own time and hospital policies

## Slide 20
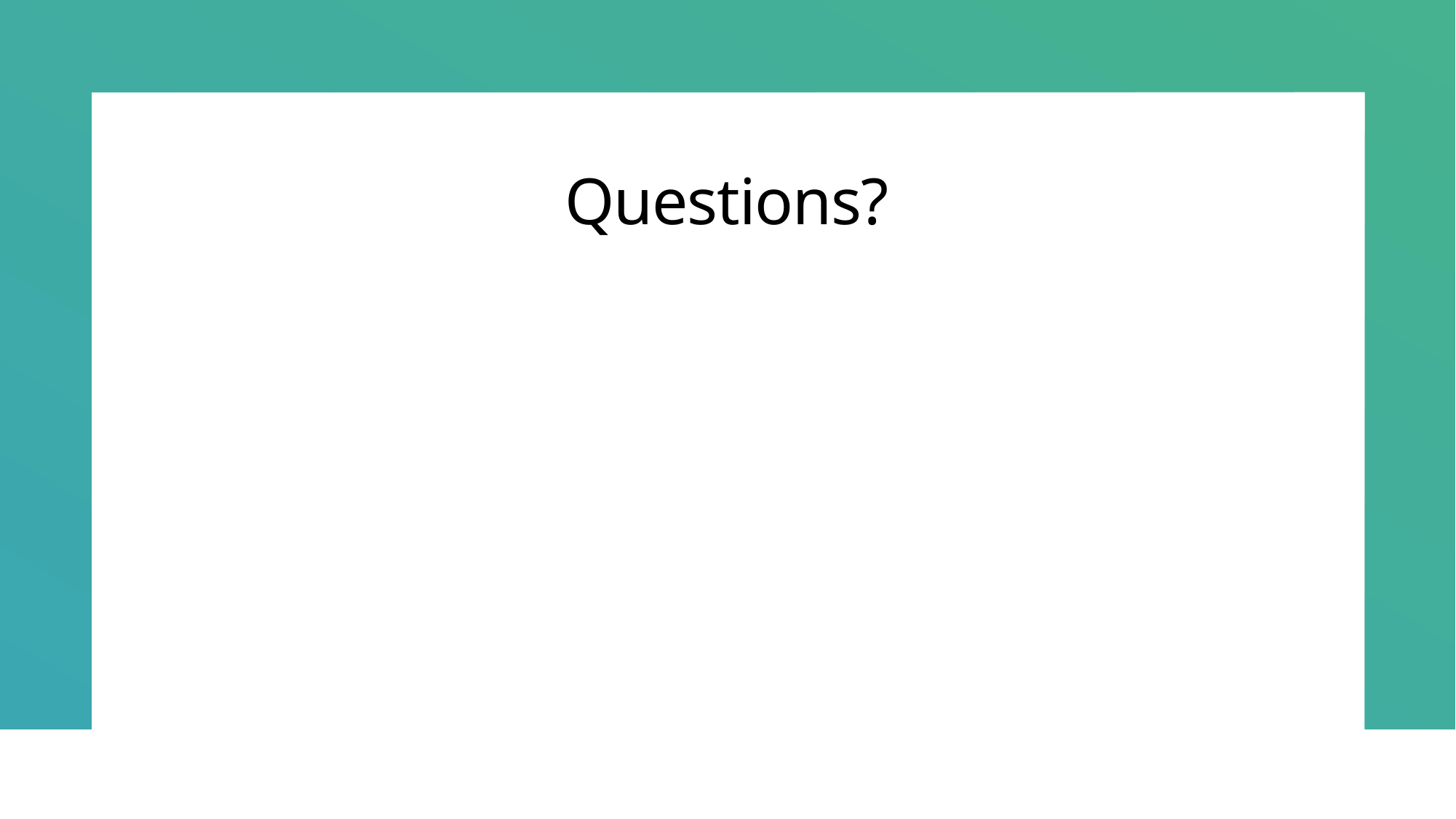

# Questions?

## Slide 21
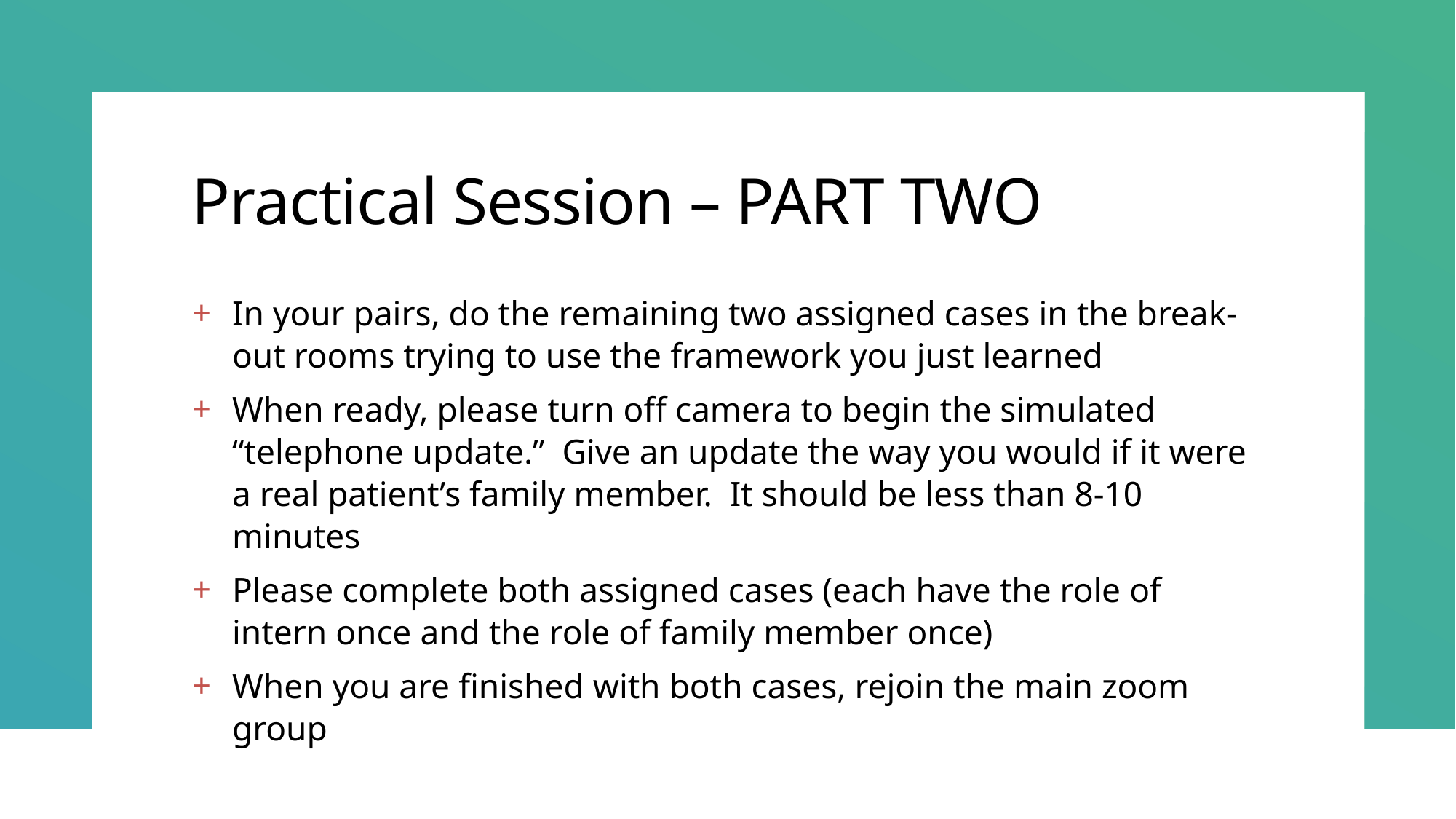

# Practical Session – PART TWO
In your pairs, do the remaining two assigned cases in the break-out rooms trying to use the framework you just learned
When ready, please turn off camera to begin the simulated “telephone update.” Give an update the way you would if it were a real patient’s family member. It should be less than 8-10 minutes
Please complete both assigned cases (each have the role of intern once and the role of family member once)
When you are finished with both cases, rejoin the main zoom group

## Slide 22
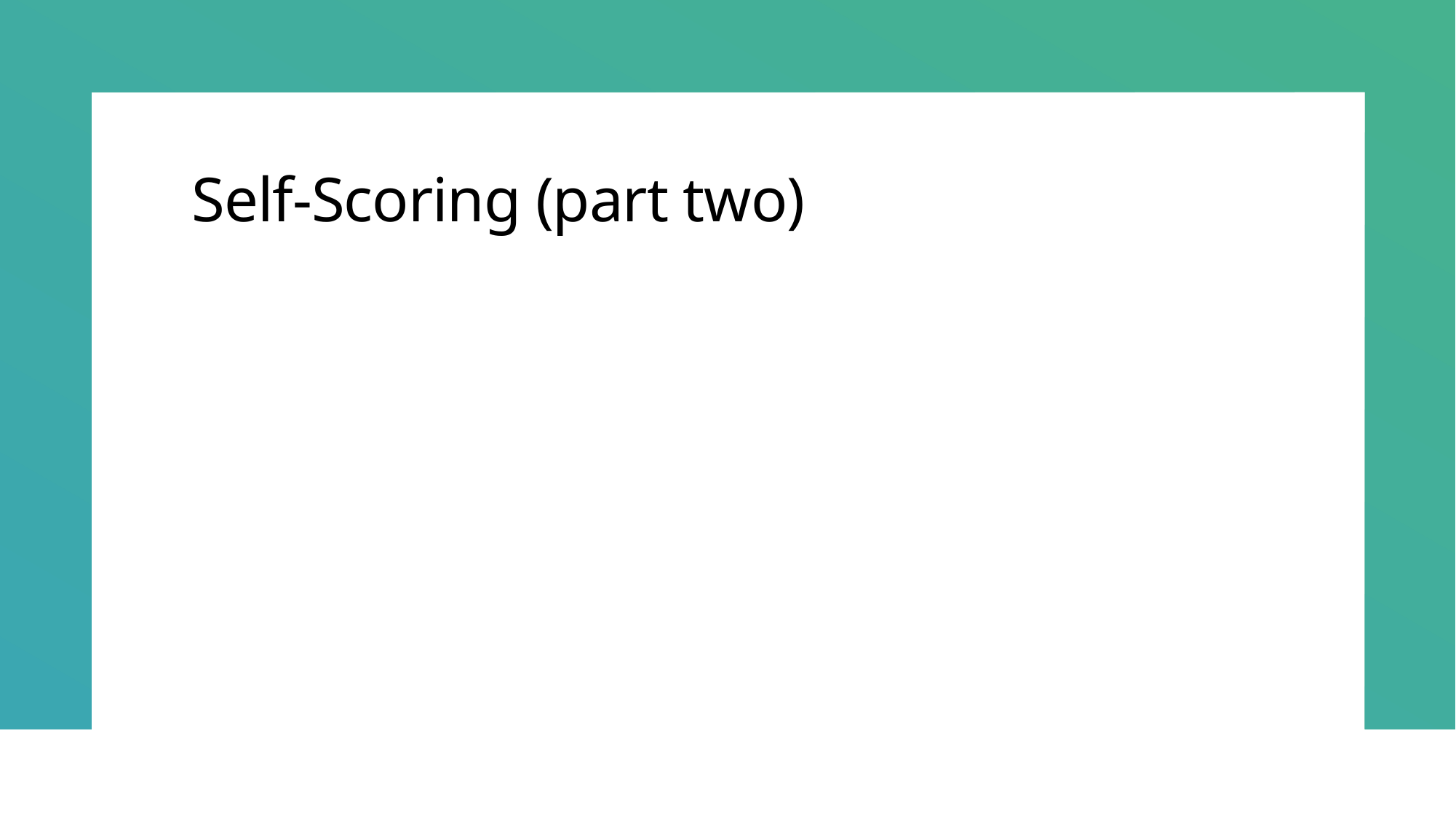

# Self-Scoring (part two)

## Slide 23
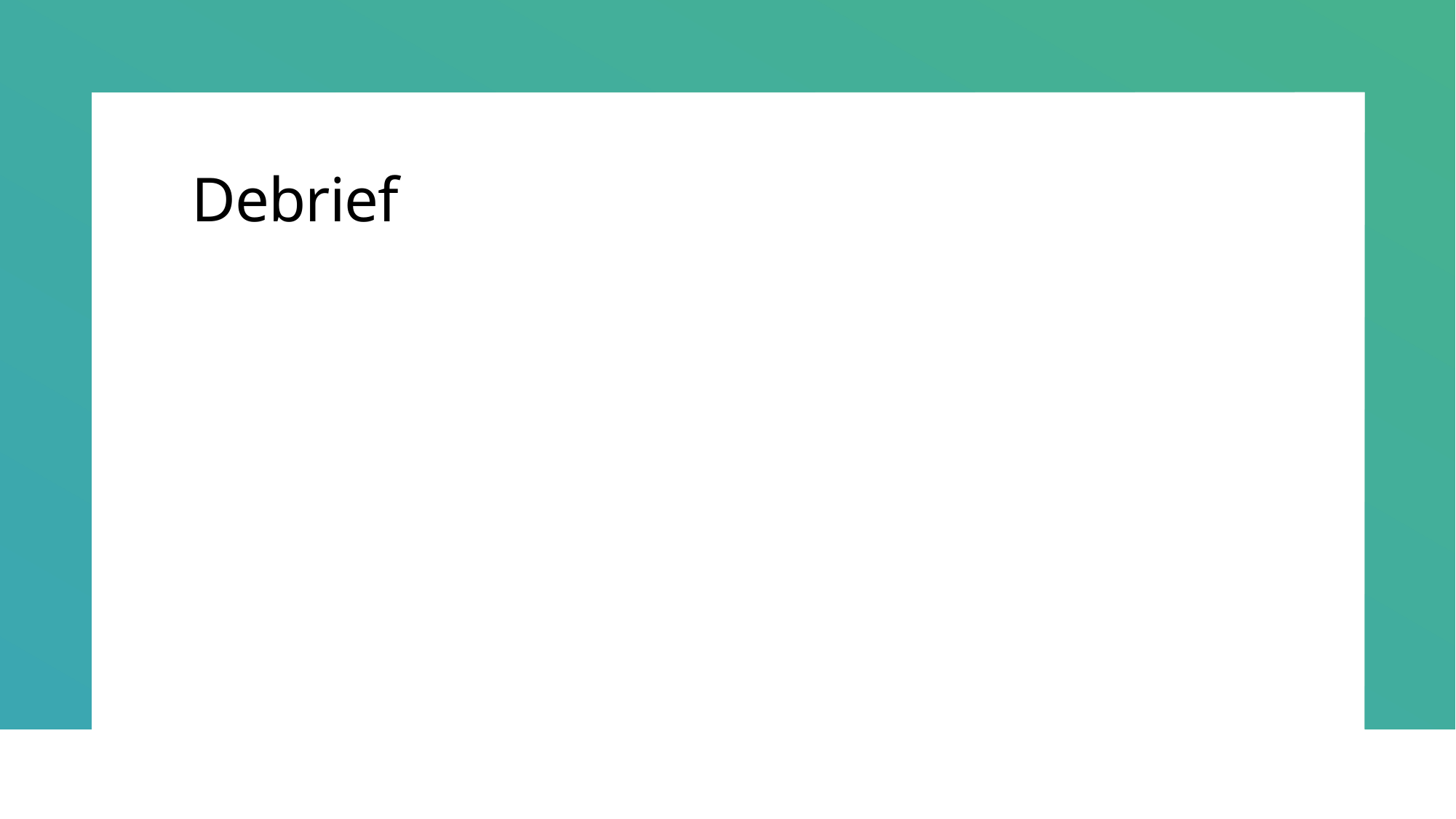

# Debrief
